# Pseudo-value regression trees

**DOI:** 10.1007/s10985-024-09618-x

**Published:** 2024-02-25

**Authors:** Alina Schenk, Moritz Berger, Matthias Schmid

**Affiliations:** Institute of Medical Biometry, Informatics and Epidemiology, Medical Faculty, University of Bonn, Bonn, Germany

**Keywords:** Gradient boosting, Interactions, Model trees, Pseudo-values, Survival probabilities, 62N01, 62N02, 62P10

## Abstract

This paper presents a semi-parametric modeling technique for estimating the survival function from a set of right-censored time-to-event data. Our method, named pseudo-value regression trees (PRT), is based on the pseudo-value regression framework, modeling individual-specific survival probabilities by computing pseudo-values and relating them to a set of covariates. The standard approach to pseudo-value regression is to fit a main-effects model using generalized estimating equations (GEE). PRT extend this approach by building a multivariate regression tree with pseudo-value outcome and by successively fitting a set of regularized additive models to the data in the nodes of the tree. Due to the combination of tree learning and additive modeling, PRT are able to perform variable selection and to identify relevant interactions between the covariates, thereby addressing several limitations of the standard GEE approach. In addition, PRT include time-dependent effects in the node-wise models. Interpretability of the PRT fits is ensured by controlling the tree depth. Based on the results of two simulation studies, we investigate the properties of the PRT method and compare it to several alternative modeling techniques. Furthermore, we illustrate PRT by analyzing survival in 3,652 patients enrolled for a randomized study on primary invasive breast cancer.

**Supplementary Information:**

The online version contains supplementary material available at 10.1007/s10985-024-09618-x.

## Introduction

The estimation of individual-specific survival probabilities is a common task in time-to-event analysis. A plethora of methods has been developed to address this issue, including, among many other approaches, group-wise Kaplan-Meier estimation, Cox regression (Cox 1972), parametric accelerated failure time models (Kalbfleisch and Prentice 2002), and inverse-probability-of-censoring-(IPC)-weighted regression models (Molinaro et al. 2004). Although these approaches are widely used in many disciplines, they often rely on restrictive assumptions limiting their utility. A notable example is the Cox regression model, which requires careful interpretation when the proportional hazards assumption is violated (e.g. Stensrud and Hernán 2020). Similarly, parametric accelerated failure time models may produce invalid results when the underlying distributional assumptions are not met, and IPC-based methods are biased if the working model for the censoring process is misspecified (van der Laan and Robins 2003). Invalid findings may also occur when the complexity of the data-generating process is not fully captured by the model, for instance when relevant covariates are excluded or when interactions between covariates remain undetected (e.g. Vatcheva et al. 2015). In some cases, model misspecification can be avoided by employing methods from the machine learning field (e.g. survival random forests, Ishwaran et al. 2008, or deep neural networks, Lee et al. 2018; Zhao and Feng 2020); however, application of these techniques is often infeasible due to small sample sizes or limitations in the interpretability of the estimated predictor-response relationships. For these reasons, it remains a challenging task to specify time-to-event models yielding accurate and interpretable estimates of individual-specific survival probabilities.

In this paper we propose a novel model building technique named *pseudo-value regression trees* (PRT). Our method is based on pseudo-value regression (Klein and Andersen 2005), which provides a direct modeling framework to estimate the survival function from a set of right-censored time-to-event data. Unlike Cox regression, pseudo-value regression is not based on a statistical model for the hazard function (from which the survival function can subsequently be derived by application of a suitable transformation); instead, it defines a direct link between the survival function and the covariate values on a grid of pre-specified time points $t_1, \ldots , t_K$. Usually, *K* is set to a moderate number, e.g. $K=5$ or $K = 10$ (see Andersen and Pohar 2010). Given data from a set of *n* independent individuals with survival times $T_i \in \mathbb {R}^+$ and time-independent baseline covariates $X_i \in \mathbb {R}^p$, $i=1,\ldots , n$, the key idea of pseudo-value regression is to approximate the survival probabilities $S(t_k |X_i) = \text{ P }(T_i> t_k|X_i) = \text{ E }[\mathbbm {1}_{\lbrace {T_i > t_k\rbrace }}|X_i]$, $k=1,\ldots , K$, by a set of jackknife *pseudo-values*. The latter are defined as

1 $$\begin{aligned} \hat{\theta }_{i}(t_k) = n \cdot \hat{S}_{\text {KM}}(t_k) - (n-1)\cdot \hat{S}_{\text {KM}}(t_k)^{-i} \,, \ \ \ i=1,\ldots , n\,, \end{aligned}$$

where $\hat{S}_{\text {KM}}(t_k)$ and $\hat{S}_{\text {KM}}(t_k)^{-i}$ denote the Kaplan-Meier estimators based on the complete data and the reduced data (without individual *i*), respectively. Since it can be shown that $\text{ E }[\hat{\theta }_i (t_k)|X_i] {\mathop {\longrightarrow }\limits ^{P}}\text{ E }[\mathbbm {1}_{\lbrace {T_i > t_k\rbrace }} |X_i]$ as $n\rightarrow \infty$ (provided that the censoring mechanism is independent of the event times and the covariates, Graw et al. 2009; Overgaard et al. 2017), consistent estimates of $S(t_k |X_i)$ can be obtained by fitting a statistical model that regresses the pseudo-values on the covariates (Andersen and Pohar 2010). Unlike IPC-based methods, which often discard the covariate information of censored individuals when used in combination with (weighted) regression techniques (Molinaro et al. 2004), pseudo-value regression is based on a “pseudo” complete data set that includes all available values $X_1,\ldots , X_n$ in the estimation equation (Andersen and Pohar 2010).

The standard approach to fit a pseudo-value model is to specify a monotonically increasing link function $g(\cdot )$ and to use $\hat{\theta }_{i}(t_k)$ as outcome variable in the regression model

2 $$\begin{aligned} g\left( S(t_k \vert X_i) \right) = g\left( \text{ E }\left[ \mathbbm {1}_{\lbrace {T_i > t_k\rbrace }} \vert X_i \right] \right) = \alpha _k + \gamma ^T X_{i} \,, \ \ \ k=1,\ldots , K\,, \end{aligned}$$

where $(\alpha _1, \ldots , \alpha _K, \gamma ^\top )^\top \in \mathbb {R}^{K+p}$ is a vector of unknown coefficients. Estimation of the coefficients is usually based on generalized estimating equations (GEE, Liang and Zeger 1986), setting $g(\cdot )$ equal to the complementary log-log link function (Andersen et al. 2003; Andersen and Pohar 2010). While the GEE approach accounts for possible dependencies between the pseudo-values $\hat{\theta }_{i}(t_1),\ldots , \hat{\theta }_{i}(t_K)$ obtained from the same individual, it is limited by the restrictive definition of the predictor $\eta _{ik} = \alpha _k + \gamma ^T X_{i}$. In particular, $\eta _{ik}$ does not allow for modeling time-dependent effects (since $\gamma$ is assumed to be constant in time), and it is restricted to modeling main covariate effects only. Although more flexible effect terms (representing e.g. interactions with time or between the covariates) could be included in (2), we are not aware of any algorithm to identify these terms in a data-driven way. On the other hand, pre-specification of the interaction terms is often infeasible, as it would require detailed knowledge on the, usually hidden, interaction structure in the data-generating process. Another limitation of the standard regression model in (2) is that the intercept terms $\alpha _1,\ldots , \alpha _K$ (representing the “baseline” risk function) are estimated in an unrestricted fashion. As a consequence, the fitted survival probabilities are not guaranteed to decrease with time.

To address these limitations, we extend the standard model in (2) by a semi-parametric approach for the estimation of survival probabilities via pseudo-value regression. Our proposed PRT method is inspired by *logistic model trees* (LMT, Landwehr et al. 2005), which is a popular classification method combining the strengths of tree learning and binary regression by fitting a series of regularized logistic models to the data in the nodes of a classification tree. In order to adapt LMT to pseudo-value regression, we propose to replace the classification tree by a multivariate conditional inference tree (Hothorn et al. 2006) and to use a novel GEE-type optimization criterion for modeling the pseudo-values in the nodes. The proposed PRT method does not require pre-specification of any main or interaction effects, neither among the covariates nor between the covariates and time.

Briefly, the PRT method is characterized by the following steps: First, in order to identify the most important interactions between the covariates, we build a multivariate conditional inference tree (Hothorn et al. 2006) using the pseudo-values as *K*-dimensional continuous response variable. In the second step, we apply a gradient boosting algorithm with linear base-learners (Bühlmann and Hothorn 2007; Hofner et al. 2014) to the data in each node of the tree. Our node-wise boosting algorithm is based on the aforementioned GEE-type optimization criterion, including a pre-specified link function to ensure that survival probability estimates are bounded between 0 and 1. Following the idea of LMT, the fitted values of boosting models in higher-level nodes are used as offset values to refine models in lower-level nodes, leading to the stabilization of estimates along single paths. In each node, the fitting of boosting models is stopped early, enabling the selection of relevant covariates. Furthermore, to model interactions between time and the covariates used to build the tree, we include a time-dependent monotonic base-learner (Hofner et al. 2011) in each node-wise model. This base-learner also ensures that survival probability estimates decrease with time.

The result of our model building technique is a set of pseudo-value regression models, each corresponding to a single path from the root node to a terminal node of the conditional inference tree. Due to the combination of tree learning and model-based boosting, the node-wise models include a mixture of interaction and time-dependent effects, all of which are identified in a data-driven way. Furthermore, PRT guarantees interpretability of the node-wise boosting fits by additively combining linear and monotonic base-learners (Hofner et al. 2014). Estimates of individual survival probabilities are obtained by dropping the covariate values down the tree and by evaluating the pseudo-value regression model in the respective terminal node.

The rest of the paper is organized as follows: In Sect. 2.1, we will start with the definition and properties of pseudo-values, including a description of the standard GEE approach for pseudo-value regression. Section 2.2 provides a brief introduction to logistic model trees (Landwehr et al. 2005). Section 3 contains a detailed description of the PRT method, including definitions of the multivariate recursive partitioning and model-based boosting techniques. In Sect. 4 we will present two simulation studies investigating the properties of the PRT method. Furthermore, we will present a comparison to established methods for survival probability estimation. In Sect. 5, we will apply the PRT method to data from the randomized phase III SUCCESS-A trial (de Gregorio et al. 2020), demonstrating that PRT are able to identify subgroups and predictors of disease-free survival in patients with non-metastatic breast cancer. The main findings of the paper are summarized and discussed in Sect. 6, along with a brief overview and discussion of related approaches. Further results and illustrations, as well as details on the implementation of the PRT method, are provided in the Supplementary Material.

## Prerequisites

### Pseudo-values for survival probability estimation

Consider a set of *n* independent individuals with survival times $T_i$ and covariate values $X_i = (X_{i1}, \ldots , X_{ip})^\top$, $i = 1, \ldots , n$, that are subject to right-censoring. Denote the censoring times and the observed survival times by $C_i$ and $\tilde{T}_i = \min (T_i, C_i)$, respectively. The status variable $\Delta _i$ indicates whether the *i*-th individual is censored ($\Delta _i = 0$) or whether the event of interest has been observed ($\Delta _i = 1$). Following Graw et al. (2009), we assume that the censoring times are independent of both the covariates and the event times.

The aim of pseudo-value regression is to model the expectation of a function $\psi (T_i)$ conditional on $X_i$ (Andersen and Pohar 2010). A special case, which will be considered in this paper, is the conditional survival probability $S(t_k|X_i) = \text{ E }[\mathbbm {1}_{\lbrace {T_i > t_k\rbrace }} \vert X_i]$ for time points $t_k$, $k =1, \ldots , K$, with $\psi (T_i) = \mathbbm {1}_{\lbrace {T_i > t_k \rbrace }}$. In order to fit a regression model for $\text{ E }\left[ \psi (T_i) \vert X_i \right] = \text{ E }[\mathbbm {1}_{\lbrace {T_i > t_k\rbrace }} \vert X_i]$, knowledge about the values $\mathbbm {1}_{\lbrace {T_i > t_k \rbrace }}$ is required. In the absence of censoring, $\mathbbm {1}_{\lbrace {{T}_i > t_k\rbrace }}$ is observable for all individuals: As $T_i = \tilde{T}_i$, it is simply given by $\mathbbm {1}_{\lbrace {\tilde{T}_i > t_k\rbrace }}$. In this case, the Kaplan-Meier estimator is precisely one minus the empirical cumulative distribution function, implying that the pseudo-value $\hat{\theta }_i (t_k)$ (as defined in (1)) coincides with $\mathbbm {1}_{\lbrace {\tilde{T}_i > t_k\rbrace }}$. In the presence of censoring, $\mathbbm {1}_{\lbrace {T_i > t_k\rbrace }}$ is not observable for all individuals; in this case the idea is to replace $\mathbbm {1}_{\lbrace {T_i > t_k\rbrace }}$ by pseudo-values for both, censored and uncensored individuals (Andersen and Pohar 2010).

**Fig. 1:**
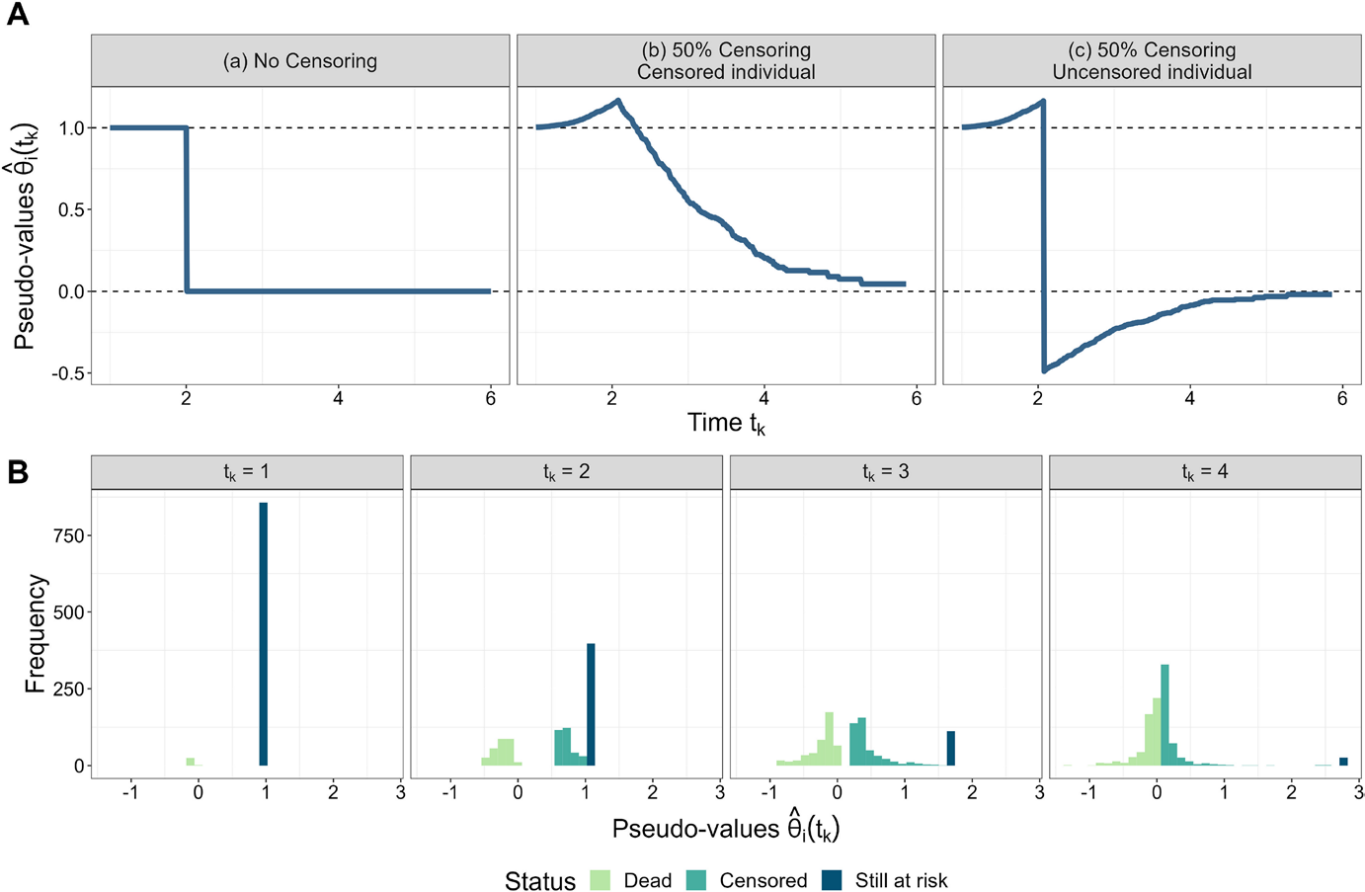
**A** Illustration of pseudo-values obtained from two data sets with $n = 1000$ individuals each ($0 \le t_k \le 6$, adapted from Andersen and Pohar 2010). Panel (a) refers to an individual with $\tilde{T}_i = T_i = 2$ in a censoring-free data set, whereas the other panels refer to a censored individual with $\tilde{T}_i = 2, \Delta _i = 0$ (Panel (b)) and an uncensored individual with $\tilde{T}_i = 2, \Delta _i = 1$ (Panel (c)) in a data set with $50\%$ right-censored survival times. In the censoring-free scenario (a), the pseudo-value at time $t_k$ is simply a binary function indicating whether the individual is still event-free at $t_k$ ($\hat{\theta }_i(t_k)=1$) or not ($\hat{\theta }_i(t_k)=0$). In the scenario with $50\%$ censoring, the individuals in (b) and (c) have exactly the same pseudo-values up to their common observed survival time ($\tilde{T}_i=2$), showing a monotonically increasing pattern. After $\tilde{T}_i=2$, the pseudo-values of the two individuals differ: While the censoring of the individual in (b) caused $\hat{\theta }_i(t_k)$ to become monotonically decreasing after $\tilde{T}_i=2$, the observed event in (c) caused $\hat{\theta }_i(t_k)$ to drop to a negative value at $\tilde{T}_i=2$ and to increase afterwards. **B** Histograms of pseudo-values at different time points in the data set with $50\%$ right-censored survival times from (A). The colors indicate the status of the individuals at the respective time points (dead, censored, still at risk). Pseudo-values of individuals that were observed to experience the event of interest before $t_k$ are negative, whereas pseudo-values are $\ge 1$ in individuals that are still at risk at $t_k$. Obviously, the distribution of the pseudo-values is strongly dependent on both the censoring pattern and the time point of interest

Figure 1 (A) provides an illustration of pseudo-values in a censoring-free data set (left panel) and in a set of right-censored data (middle and right panels, adapted from Andersen and Pohar 2010). The figure shows that the values $\hat{\theta }_i(t_k)$ are not bounded between 0 and 1 in the presence of censoring. In particular, when focusing on single time points (Fig. 1 (B)), it appears hard to approximate the empirical distribution of pseudo-values by a parametric distribution (as it strongly depends on both the time point and the censoring pattern).

As outlined in Sect. 1, the standard approach to pseudo-value regression is to use the unconditional values $\hat{\theta }_i (t_k)$ as outcome variable in a GEE model of the form (2). Defining the *response function* by $h(\cdot ):= g^{-1}(\cdot )$, it is convenient to re-write Equation (2) as

3 $$\begin{aligned} S(t_k|X_i)&= \text{ E }\left[ \mathbbm {1}_{\lbrace {T_i > t_k\rbrace }} \vert X_i \right] = g^{-1}(\beta ^T X_{i,k}) = h(\beta ^T X_{i,k}) \, , \end{aligned}$$

where the augmented covariate vector $X_{i,k} = (0,\ldots ,0,1,0,\ldots ,0, X_i^\top )^\top \in \mathbb {R}^{K+p}$ contains an additional set of *K* binary indicators that are all zero except for the *k*-th one. The coefficient vector $\beta = (\alpha _1,\ldots , \alpha _K, \gamma ^\top )^\top \in \mathbb {R}^{K+p}$ comprises both the baseline risk function and the covariate effects. Common choices for the response function are $h(\beta ^T X_{i,k}) = \exp (\beta ^T X_{i,k}) / (1+\exp (\beta ^T X_{i,k}))$ (corresponding to the logit link) and $h(\beta ^T X_{i,k}) = 1 - \exp ( -\exp (-\beta ^T X_{i,k}))$ (corresponding to the complementary log-log link, Klein and Andersen 2005). Both functions ensure that the survival probabilities $S(t_k|X_i)$ in (3) are bounded between 0 and 1.

Denoting $h(\beta ^TX_{i,.}) = (h(\beta ^TX_{i,1}), \ldots , h(\beta ^TX_{i,K}))^T$ and $\hat{\theta }_i = (\hat{\theta }_i (t_1),\ldots , \hat{\theta }_i (t_K))^\top$, the GEE estimate of $\beta$ is given by the solution to

4 $$\begin{aligned} \sum \limits _i \Big \{\frac{\partial }{\partial \beta }\, h(\beta ^TX_{i,.}) \Big \}^T V_i^{-1} \Big \{\hat{\theta }_i - h(\beta ^TX_{i,.})\Big \} = 0 \, , \end{aligned}$$

where $V_i \in \mathbb {R}^{K \times K}$ defines a working covariance matrix accounting for possible dependencies between pseudo-values obtained from the same individual. In practice, $V_i$ is often set to a diagonal matrix (corresponding to an independent correlation structure), as Klein and Andersen (2005) found no advantage of using more complex versions. As shown by Graw et al. (2009), solving (4) yields a consistent ($n\rightarrow \infty$) and asymptotically normal estimator for $\beta$, provided that the model is specified correctly.

### Logistic model trees (LMT)

To address the issues described in Sect. 1 and to extend Model (2) to more complex situations containing interactions and time-dependent effects, we propose to build pseudo-value regression models using an adaptation of the LMT method (Landwehr et al. 2005). Originally, LMT have been proposed to develop classification models with a binary outcome. The method consists of two main steps: First, relevant subgroups and interactions are detected by growing a classification tree on the complete data set (see Sect. 3.1 for details on the tree construction). Second, binary logistic regression models are fitted to the data in each node of the tree, resulting in the estimation of covariate-dependent (node-wise) class probabilities. Unlike earlier approaches to combining tree learning with regression modeling (Quinlan 1992), Landwehr et al. did not fit standard regression models (based on maximum likelihood estimation) but used the LogitBoost method with simple regression functions (Friedman et al. 2000) to build regularized main-effects logistic models (see Sect. 3.2 for details on boosting). Of note, LogitBoost avoids overfitting the data by identifying subsets of the covariates that are most relevant to the node-wise fits. As a consequence, the LMT method performs variable selection at two levels: First, the classification tree selects the covariates that are most relevant to creating subgroups of the data; second, LogitBoost selects the covariates that are most relevant to the node-wise models.

An important characteristic of LMT is the successive refinement of the boosting fits in each tree level, which is achieved by node-wise updates of the LogitBoost coefficients: Starting at the root node of the tree and descending down to the terminal nodes, the LogitBoost coefficients in each daughter node are constructed as updated versions of the coefficients in the respective parent node (Landwehr et al. 2005). Thus, information from higher-level nodes (closer to the root) is incorporated in the models at lower levels, leading to a stabilization of the model fits in the terminal nodes. The estimated class probability for an individual is obtained by dropping the respective covariate values down to a terminal node and evaluating the logistic model associated with that node.

As LMT are a combination of logistic regression and tree learning, they are considerably more flexible than either of the two methods alone, covering both simple main-effects logistic models and standard classification trees as special cases. More specifically, a classification tree of depth 0 (no splits) with a LogitBoost procedure in the root node represents the simple (main-effects-only) logistic model whereas a classification tree of any depth $>0$ and no covariates selected by LogitBoost is equivalent to a standard classification tree (Landwehr et al. 2005).

## Pseudo-value regression trees (PRT)

Given the limitations of the standard GEE approach, and considering the flexibility of LMT in dealing with complex interaction structures, we propose to build pseudo-value regression models by extending the LMT methodology to the estimation of survival probabilities. Briefly, the idea of our *pseudo-value regression trees (PRT)* approach is to replace the binary classification tree by a conditional inference tree with multivariate pseudo-value outcome (accounting for possible dependencies between pseudo-values from the same individual, Sect. 3.1), to replace LogitBoost by a component-wise gradient boosting algorithm (including a time-dependent monotonic base-learner and a novel GEE-type optimization criterion, Sect. 3.2), and to use the successively refined boosting models for the estimation of individual-specific survival probabilities (Sect. 3.3).

### Tree building

The first step of PRT is to grow a regression tree on all available data, replacing the binary outcome of LMT by the pseudo-values $\hat{\theta }_i (t_k) \in \mathbb {R}$, $i=1,\ldots , n$, $k=1,\ldots , K$. Generally, the PRT method is not restricted to a specific algorithm for tree building, see e.g. Greenwell (2022) for an overview of the many available options. However, one needs to account for possible dependencies between the pseudo-values obtained for the same individual. To address this issue, we consider the *multivariate conditional inference framework* (Hothorn et al. 2006), which allows for building regression trees with a *K*-dimensional outcome.

#### Conditional inference trees

The general idea of tree building is to derive local estimates of the outcome variable by partitioning the covariate space into a set of mutually exclusive subspaces (Breiman et al. 1984; Hothorn et al. 2006; Greenwell 2022). Starting at the *root node* of the tree (comprising all individuals), tree building is done recursively by applying a set of decision rules to the available data. Usually, the decision rules are binary, implying that each node is followed by two *daughter nodes* (each containing a subgroup of the individuals). Tree building is terminated when a pre-defined stopping criterion is reached, resulting in a set of *terminal nodes* from which the local estimates of the outcome are derived. In case of PRT, the local estimates are given by the node-wise boosting fits (see Sect. 3.3).

During tree building, all decision rules are derived locally from the individuals in the respective node. Each rule is characterized by a *split variable* that is selected in a data-driven way from the covariate set. In case of a continuous split variable $x^*$, the decision rule is defined by $x^* > \xi$ vs. $x^* \le \xi$, where $\xi \in \mathbb {R}$ is a threshold estimated from the data. In case of a categorical split variable, the decision rule is obtained by dividing the set of categories into two mutually exclusive subsets.

Within this framework, the conditional inference approach (Hothorn et al. 2006) is a method for tree construction that accounts for the distributional properties of the covariates (thereby avoiding a selection bias towards covariates with many possible splits). Decision rules are derived as follows: Given a node with individuals $\mathcal {N} \subseteq \{1,\ldots , n\}$ and data $\mathcal {L} = \lbrace (\hat{\theta }_{i} (t_1), \ldots , \hat{\theta }_{i} (t_K), X_{i1},\ldots , X_{ip})$, $i \in \mathcal {N} \rbrace$, the first step is to determine the covariate showing the strongest association with the outcome variable. In PRT, this is done by evaluating the generalized correlation coefficients

5 $$\begin{aligned} T_j(\mathcal {L}) = \text{ vec }\left( \sum _{i \in \mathcal {N}} \tilde{g}_j (X_{ij}) \cdot (\hat{\theta }_i (t_1), \ldots , \hat{\theta }_i (t_K))^\top \right) \in \mathbb {R}^{\tilde{p}_j \times K} \, , \ \ \ j=1,\ldots , p \, , \end{aligned}$$

where $\tilde{g}_j (\cdot ) \in \mathbb {R}^{\tilde{p}_j}$, $j=1,\ldots , p$, is a set of transformation functions depending on the measurement scales of the covariates. For the purposes of PRT, we set $\tilde{g}_j (X_{ij}) = X_{ij}$ if the *j*-th covariate is measured on a continuous scale. For unordered and ordered factors, the functions $\tilde{g}_j (X_{ij})$ are given by a set of dummy variables or some other coding. Next, the elements of $T_j(\mathcal {L})$ are standardized (assuming conditional independence of the covariates and the outcome, see Hothorn et al. 2006) and transformed using the absolute value function. By this, the standardized and transformed elements of $T_j(\mathcal {L})$ can be interpreted as absolute correlations between the *j*-th covariate and each of the *K* pseudo-value elements. Specifically, a separate correlation coefficient is computed at each $t_k$, $k \in \{1,\ldots , K\}$, so that the dependency between the pseudo-values and time (which is possibly non-monotonic, see Fig. 1) does not affect these calculations. For each *j*, the maximum value of the absolute correlations is then used to measure the association between the *j*-th covariate and the *K*-dimensional pseudo-value outcome and to test the null hypothesis of independence. Altogether, there are *p* maximum values, resulting in *p* hypothesis tests. Again, by definition, each of the *p* maximum values refers to only one time point $t_k$, $k \in \{1, \ldots , K\}$, so that the tree building step of PRT does not depend on the functional form of the relationship between the pseudo-value outcome and time. Using the default specification in the R package **partykit**, we employ 9,999 permutations to determine the conditional distributions of the maximum values under the null. Finally, the covariate with minimum p-value in the permutation tests is selected as split variable. By definition of this procedure, both the construction of the coefficients in (5) and the implementation of the subsequent hypothesis tests (permuting individuals instead of single pseudo-values) account for the multivariate structure of the vectors $(\hat{\theta }_{i}(t_1), \ldots , \hat{\theta }_{i}(t_K))$.

The second step is to derive the actual decision rule associated with the selected covariate. This is done by determining either a threshold $\xi$ (if the selected covariate is continuous) or a grouping of the categories (if the selected covariate is a factor), such that the daughter nodes become maximally dissimilar with respect to the outcome variables. Denoting the set of possible decision rules by $\mathcal {S}$, each decision rule $s \in \mathcal {S}$ is characterized by two mutually exclusive sets of individuals $\mathcal {N}_{\text {left},s}$ and $\mathcal {N}_{\text {right},s}$, referring to the daughter nodes. In order to determine the optimal decision rule, the idea is to maximize

6 $$\begin{aligned} \max _{k \in \{1,\ldots , K\}} \, \left| \frac{\sum _{i \in \mathcal {N}} \mathbbm {1}_{ \{ i \in \mathcal {N}_{\text {right},s}\}} \cdot \hat{\theta }_{i} (t_k) - \mu _{k,s}}{\sigma _{k,s} } \right| \end{aligned}$$

over all decision rules $s \in \mathcal {S}$, where $\mu _{k,s}$ and $\sigma _{k,s}$, denote the conditional means and standard deviations, respectively, of $\sum _{{i}\in \mathcal {N}} \mathbbm {1}_{\{ {i \in \mathcal {N}_{\text {right},s}}\}} \cdot \hat{\theta }_{i} (t_k)$, $k=1,\ldots , K$ (computed in the same way as above, cf. Hothorn et al. 2006). By definition, the coefficients in (6) measure the association between node membership and the outcome values; hence, maximizing (6) ensures that the sets of individuals in the daughter nodes become maximally dissimilar with respect to the outcome. Note that each of the decision rules $s\in \mathcal {S}$ depends on the selected covariate; for ease of notation we did not indicate this dependency in (6).

#### Tuning of the tree

Generally, the partitioning steps described in Sect. 3.1.1 could be applied until each terminal node contains exactly one individual. In case of PRT, this situation would be clearly undesirable, as a large number of terminal nodes would compromise the interpretability of the tree. Furthermore, the tree tends to overfit the data if the node sizes are too small, leading to numerical issues with the fitting of the boosting models.

To ensure interpretability of the PRT model, we propose to fix the depth *D* of the regression tree at a small number. In our experiments (Sects. 4.1 and 4.2) we used $D \le 5$, noting that $D=5$ (referring to five-way interactions) is already a large value regarding interpretability. Also note that, in some cases, tree building could be terminated before the value *D* is reached. For instance, the current implementation of the conditional inference tree method in the R package **partykit** stops tree building if all p-values of the permutation tests are larger than a pre-specified threshold. For the purposes of PRT, we set this threshold to 0.05. In addition to restricting the depth of the tree, we require a pre-specified minimum number of observations in each terminal node. In our experiments we set this number to $5 \cdot K$, i.e. to five times the number of time points.

### Component-wise gradient boosting

After having grown the regression tree, the next step is to apply a gradient boosting procedure to the data in each node. Here we propose to consider *component-wise gradient boosting*, as described in Bühlmann and Hothorn (2007) and Hofner et al. (2014).

For gradient boosting it is convenient to organize the data in long format: Since the pseudo-values differ between time points, the idea is to create an augmented data matrix representing each individual by *K* rows (one per time point, resulting in an overall number of $n \cdot K$ rows). Furthermore, the augmented data matrix includes an additional ID column, as well as a continuous covariate containing the time values $t_1,\ldots , t_K$. In the following, we will refer to the rows of the augmented data matrix as *observations* (denoted by $\tilde{\imath } \in \lbrace {1, \ldots , n\cdot K\rbrace }$), in contrast to individuals. Table 1 presents the augmented data of two exemplary individuals.

**Table 1:** Augmented data of two exemplary individuals, assuming three time points $t_1 = 0.3$, $t_2 = 1.5$, and $t_3 = 3.8$. Each individual is represented by $K=3$ observations (= rows), each referring to one of the time points. The *ID* column is a factor identifying the individuals, and the covariate values (which are assumed to be time-independent) are replicated *K* times each (columns $x_1,\ldots , x_p$). The $x_0$ column refers to an intercept term that is needed for technical reasons in the gradient boosting algorithm

ID	Time	Pseudo-value	$x_0$	$x_1$	.	$x_p$
1	0.3	1.003	1	0.46	.	− 0.27
1	1.5	0.805	1	0.46	.	− 0.27
1	3.8	0.359	1	0.46	.	− 0.27
2	0.3	1.003	1	− 0.18	.	0.14
2	1.5	1.141	1	− 0.18	.	0.14
2	3.8	− 0.822	1	− 0.18	.	0.14

#### Details on the algorithm

We first describe the procedure that is applied locally to the data in each node. Given a node with $\tilde{n}$ individuals and $\tilde{n}\cdot K$ observations (denoted by $\mathcal {M} \subseteq \{1, \ldots , n\cdot K \}$), the input of the component-wise gradient boosting procedure is an augmented data set of the form $\{ (\hat{\theta }_{\tilde{\imath }}, t_{\tilde{\imath }}, X_{\tilde{\imath } 0}, X_{\tilde{\imath } 1}, \ldots , X_{\tilde{\imath } p})$, $\tilde{\imath } \in \mathcal {M} \}$, where $\hat{\theta }_{\tilde{\imath }}$ and $t_{\tilde{\imath }}$ refer to the pseudo-values and the time points, respectively, of the $\tilde{\imath }$-th observation (*Pseudo-value* and *Time* columns in Table 1). Correspondingly, the values $X_{\tilde{\imath } 0}, X_{\tilde{\imath } 1}, \ldots , X_{\tilde{\imath } p}$ refer to the $x_0, x_1, \ldots , x_p$ columns in Table 1.

The aim of gradient boosting is to estimate an “optimal” prediction function $f^*\in \mathbb {R}$ by minimizing the empirical risk function $\mathcal {R} = \sum _{\tilde{\imath } \in \mathcal {M}} \rho (\hat{\theta }_{\tilde{\imath }}, f_{\tilde{\imath }})$ over the vector $f = \{ f_{\tilde{\imath }} \}_{\tilde{\imath } \in \mathcal {M}}= \{ f(\mathcal {X}_{\tilde{\imath }}) \}_{\tilde{\imath } \in \mathcal {M}}$, where $\mathcal {X}_{\tilde{\imath }}$ is a subset of $\{ t_{\tilde{\imath }}, X_{\tilde{\imath } 0}, X_{\tilde{\imath } 1}, \ldots , X_{\tilde{\imath } p}\}$ and $\rho \in \mathbb {R}$ is a loss function measuring the “deviation” between the outcome and some prediction function $f \in \mathbb {R}$. Note that $f^*$ is not required to depend on all available covariates; instead, the idea is to select the relevant covariates in a data-driven way (hence the term “component-wise”, which will be omitted in the following sections for the sake of brevity). The loss function will be described in more detail in Sect. 3.2.2.

Estimation of $f^*$ is performed in an iterative fashion. Starting with some offset values $\hat{f}^{[0]} = \{ \hat{f}^{[0]}_{\tilde{\imath }} \}_{\tilde{\imath } \in \mathcal {M}}$, the idea is to minimize the empirical risk function by repeating the following steps: (i) Compute the negative gradient vector $u^{[m]} = - \{ \partial \rho / \partial f_{\tilde{\imath }} \, (\hat{f}^{[m-1]}_{\tilde{\imath }}) \}_{\tilde{\imath } \in \mathcal {M}}$ (with *m* denoting the iteration number), (ii) relate $u^{[m]}$ to the time values and the covariates by a set of univariable regression estimators (denoted by $b_t (t_{\tilde{\imath }}), b_0 (X_{\tilde{\imath }0}), b_1(X_{\tilde{\imath }1}), \ldots , b_p(X_{\tilde{\imath }p})$ and fitted separately to the negative gradient vector $u^{[m]}$), (iii) select the regression estimator with the best fit, and (iv) update $\hat{f}^{[m]} = \hat{f}^{[m-1]} + \nu \cdot \hat{u}^{[m]}$, where $\nu$ is a step length factor and $\hat{u}^{[m]}$ is the vector of fitted values obtained from the selected regression estimator. For the purposes of PRT, we set $\nu = 0.01$. More details on the algorithm are given in Hofner et al. (2014).

Usually, the boosting algorithm is not run until convergence but “stopped early”, implying that the stopping iteration (denoted by $m_{\text {stop}}$) becomes the main tuning parameter of the algorithm (see Sect. 3.2.4). By early stopping, the estimate of $f^*$ is shrunken towards zero, with $\nu$ serving as a shrinkage factor. Importantly, as each of the regression estimators is linked to exactly one of the covariates or time, early stopping, together with the selection step in (iii), results in the selection of a subset of relevant covariates. Note that a regression estimator is not removed from the set of candidate estimators after being selected in step (iii), so that the same regression estimator (= covariate) might be selected in multiple iterations.

Generally, the specification of the regression estimators (hereinafter termed *base-learners*) determines the shape of the estimated function $\hat{f}^{[m_{\text {stop}}]}$. In the literature, many types of base-learners have been proposed, including smoothing splines and trees of various depths (Friedman 2001; Bühlmann and Yu 2003; Hofner et al. 2014). To increase the interpretability of the PRT model, we propose to specify simple linear base-learners for the covariates, implying that the estimators $b_j (X_{\tilde{\imath }j})$, $j=1,\ldots , p$, refer to a set of simple linear regression models. Following Hofner et al. (2014), we propose to exclude the intercept terms from these models and to specify a separate simple linear model $b_0(X_{\tilde{\imath }0})$ for the constant terms $X_{\tilde{\imath }0} \equiv 1$. Regarding the choice of $b_t(t_{\tilde{\imath }})$, we propose to use a P-spline estimator that is constrained to increase with time, thereby ensuring monotonicity of the baseline risk (see below). For the experiments in Sect. 4 we used the default implementation of monotonic P-splines in the R package **mboost**; details are given in Hofner et al. (2011).

With these specifications, and due to the additive updates in step (iv), the boosting fit at iteration $m_\text {stop}$ can be written as an additive combination of the covariates and time. More specifically, the estimated values of $f^*$ become equal to

7 $$\begin{aligned} \hat{f}^{[m_\textrm{stop}]}_{\tilde{\imath }} = \hat{f}^{[0]}_{\tilde{\imath }} + \sum _{j=0}^p \gamma _j X_{\tilde{\imath }j} + \alpha (t_{\tilde{\imath }}) \, , \ \ \ \tilde{\imath } = 1,\ldots , \tilde{n}\cdot K \, , \end{aligned}$$

where the intercept $\gamma _0$ and the slope coefficients $\gamma _j$, $j = 1, \ldots , p$, are defined by $\nu$ times the sum of the coefficient estimates of the simple linear models at the iterations at which the respective base-learners $b_j$, $j=0,1,\ldots ,p$, were selected. Analogously, the function $\alpha (\cdot )$ is defined by $\nu$ times the sum of the monotonic P-spline functions at the iterations at which $b_t$ was selected. Since $b_t$ is monotonically increasing in time (and since the same holds for the offset values $\hat{f}^{[0]}_{\tilde{\imath }}$, see below), the baseline risk (represented by $\hat{f}^{[0]}_{\tilde{\imath }} + \gamma _0 + \alpha (t_{\tilde{\imath }} )$) is also guaranteed to be monotonically increasing in time. This, in turn, leads to a monotonically decreasing survival function for a given set of covariate values, see Sect. 3.3.

In the preceding paragraphs we implicitly assumed that all covariates are measured on a continuous scale. Generally, base-learners for categorical covariates can be specified analogously (e.g. by linear models based on dummy variables or some other coding). Note, however, that care has to be taken when a categorical covariate is binary and when the same covariate has been selected as split variable in some higher-level node of the tree. In this case, the covariate will have zero variance, implying that the respective base-learner has to be excluded from the boosting algorithm. Similar adaptions have to be made for multi-categorical split variables.

*Remark:* We emphasize that restricting the base-learners to a set of simple main-effects models does not preclude the inclusion of interactions in the final PRT model. This is because gradient boosting is applied node-wise, introducing interactions between the split variable(s) and the variables selected by the boosting algorithm. In particular, the selection of the time base-learner $b_t$ defines a time-dependent effect of the split variable on survival. An illustration of the ability of PRT to model time-dependent effects is given in Section S3 in the supplementary material.

Having defined the node-wise boosting procedure, it remains to (i) specify the loss function $\rho (\hat{\theta }_{\tilde{\imath }},f_{\tilde{\imath }})$, (ii) define the offset values $\hat{f}^{[0]}_{\tilde{\imath }}$ in Equation (7), and (iii) conceive a strategy for the optimization of $m_\textrm{stop}$. We will elaborate on these issues in the following sections.

#### Specification of the loss function

Boosting algorithms with a continuous outcome often employ the squared error loss, implicitly assuming normality of $\hat{\theta }_{\tilde{\imath }}$ (“$L_2$ boosting“, Bühlmann and Yu 2003). In case of PRT, this assumption is clearly not appropriate, as the distribution of the pseudo-values is far from normal (see Fig. 1), and as the predicted survival probabilities are constrained to lie in the interval [0, 1]. We therefore propose to use a novel loss function defined by

8 $$\begin{aligned} \rho (\hat{\theta }_{\tilde{\imath }}, f_{\tilde{\imath }})&= \Big ( \hat{\theta }_{\tilde{\imath }} - (1-\exp (-\exp (-f_{\tilde{\imath }}))) \Big )^2 = \left( \hat{\theta }_{\tilde{\imath }} - h(f_{\tilde{\imath }}) \right) ^2 \, , \end{aligned}$$

which is inspired by the loss function underlying the GEE approach (assuming a complementary log-log link with $h(f_{\tilde{\imath }}) = 1 - \exp (-\exp (-f_{\tilde{\imath }}))$, see Sect. 2.1). The derivative of this loss function, which is needed to compute the negative gradient vector $u^{[m]}$, is derived as

9 $$\begin{aligned} \frac{\partial \rho }{\partial f_{\tilde{\imath }}}&= \, 2 \cdot \exp (-\exp (- f_{\tilde{\imath }})) \cdot \exp (- f_{\tilde{\imath }}) \cdot \Big ( \hat{\theta }_{\tilde{\imath }} - (1-\exp (-\exp (- f_{\tilde{\imath }}))) \Big ) \nonumber \\&= - 2 \cdot \frac{\partial h}{\partial f_{\tilde{\imath }}} \cdot \big ( \hat{\theta }_{\tilde{\imath }} - h(f_{\tilde{\imath }}) \big ) \, . \end{aligned}$$

Under the assumption that $V_i = \text {diag}(1,\ldots , 1) \in \mathbb {R}^{K \times K}$ (corresponding to an independent correlation structure), the derivative in (9) is equivalent to the criterion in (4).

#### Definition of the offset values

Following the original LMT approach by Landwehr et al. (2005), we propose to refine the node-wise boosting models by passing the characteristics of higher-level boosting fits down to the models in lower-level nodes. The general idea is to incorporate these characteristics in the node-specific offset values $\hat{f}^{[0]}$, also accounting for the time-dependency of the pseudo-values $\hat{\theta }_{\tilde{\imath }}$.

More specifically, given a node with $\tilde{n}$ individuals, $\tilde{n} \cdot K$ observations (denoted by $\mathcal {M} \subseteq \{1, \ldots ,$ $n \cdot K\}$), and augmented data $\{(\hat{\theta }_{\tilde{\imath }}, t_{\tilde{\imath }}, X_{\tilde{\imath }0}, X_{\tilde{\imath }1}, \ldots , X_{\tilde{\imath }p})$, $\tilde{\imath } \in \mathcal {M}$}, we define the offset value for some observation $\tilde{\imath }^* \in \mathcal {M}$ by

10 $$\begin{aligned} \hat{f}^{[0]}_{\tilde{\imath }^*} = \frac{1}{\tilde{n}} \, \sum _{\tilde{\imath } \in \mathcal {M}} \, \hat{f}_{\tilde{\imath }}^P \cdot \mathbbm {1}_{\lbrace {t_{\tilde{\imath }} = t_{\tilde{\imath }^*}\rbrace }} \,, \end{aligned}$$

where $\hat{f}_{\tilde{\imath }}^P$ denotes the fitted value of the $\tilde{\imath }$-th observation in the parent node. (Note that all observations $\tilde{\imath }\in \mathcal {M}$ contained in the current node are also part of the observations in the respective parent node.) Thus, the offset values $\hat{f}^{[0]} \in \mathbb {R}^{\tilde{n} \cdot K}$ are given by the time-dependent average of the fitted values of $\tilde{\imath } \in \mathcal {M}$ in the respective parent node. Regarding the root node (for which no parent node is available), the offset values $\hat{f}^{[0]} \in \mathbb {R}^{n \cdot K}$ are given by the time-dependent average of the pseudo-values in the complete data. Conceptually, Equation (10) implies that offset values in lower-level nodes depend on the covariates selected by the boosting models in higher-level nodes. We will elaborate on this dependency in Sect. 3.3.

*Remark:* The offset calculation described above implies that, in each node, there is a common “average“ time trend from which the node-wise boosting model starts iterating. Doing this, the re-calculation of the offset in each node corresponds to “shifts” of the node-wise time trends, followed by the addition of individual-specific effects (via the node-wise boosting models). The rationale of this approach is that the performance of boosting algorithms is known to strongly depend on the choice of a suitable offset value. Often, a good choice is the average value of the predictor (calculated from the data at hand and resulting in a common offset for all individuals, see e.g., Bühlmann and Hothorn 2007). The current implementation of PRT follows this idea. Alternatively, node-wise offset values could be calculated using the observation-wise predictions from the respective parent nodes. When developing the PRT method, we found that the use of average time-dependent offsets (as described above) resulted in better model fits than the “observation-wise” strategy, presumably because taking averages stabilized the fits (in the sense that residual variability in the parent models was better controlled). This is why we eventually decided to implement the “average” strategy in PRT.

#### Tuning of the gradient boosting models

For the original LMT method, Landwehr et al. (2005) proposed a heuristic to tune the number of LogitBoost iterations in the nodes of the classification tree. Instead of optimizing the iteration number separately in each node (“inner cross-validation”, which would have resulted in a high computational effort), the authors determined the optimal value of $m_\text {stop}$ in the root node (using cross-validation) and applied this value to all other (lower-level) boosting models as well. This approach significantly sped up the algorithm and worked surprisingly well in approximating the node-wise-optimized LMT model. On the other hand, the optimal $m_\text {stop}$ value determined in the root node is likely too large for the boosting models in the terminal nodes, as these models require more regularization due to the smaller node sizes. Also, cross-validation tends to show a high variability when the node size becomes small. The same is true for a modified strategy that would use the same $m_\text {stop}$ in all boosting models but would optimize this value across the whole tree (i.e. by minimizing the cross-validated sum of the loss values in Equation (8) computed from the predictions in the terminal nodes). Analogous to Landwehr et al. (2005), we therefore propose a heuristic that avoids the computational burden of optimizing $m_\text {stop}$ in every node of the tree. The main idea of our strategy is to optimize a single tuning parameter for the whole PRT model (in the following denoted by $m_\textrm{stop}(1)$) and to use this parameter for the calculation of the node-wise iteration numbers. More specifically, for a given value of $m_\text {stop}(1)$, we propose to determine the number of boosting iterations in some node $\mathcal {N}$ by

11 $$\begin{aligned} m_{\text {stop}}(\mathcal {N}) = \frac{\tilde{n}_{\mathcal {N}}}{n} \cdot m_{\text {stop}}(1) \, , \end{aligned}$$

where $\tilde{n}_{\mathcal {N}}$ is the number of individuals in $\mathcal {N}$. By definition, Equation (11) links the node-specific iteration numbers to the numbers of individuals contained in the respective nodes. It also implies that the tuning parameter $m_\textrm{stop}(1)$ becomes equal to the number of boosting iterations in the root node (where $\tilde{n}_{\mathcal {N}}=n$). Unlike the tuning approach of LMT, Eq. (11) does not assign the same iteration number to all nodes; instead, the node-specific values $m_{\text {stop}}(\mathcal {N})$ are forced to decrease as the tree depth increases. By this, our strategy incorporates the well-established result that boosting models applied to smaller data sets (found in lower-level nodes) require more regularization (i.e. smaller values of $m_\text {stop}$) than models applied to larger data sets (found in higher-level nodes). In line with this result, the largest number of iterations is assigned to the root node. In our experiments (Sect. 4) we determined the optimal value of $m_{\text {stop}}$ by five-fold cross-validation, minimizing the loss function (8) in the terminal nodes (averaged across all observations in the test folds).

*Remark:* In order to avoid overoptimism in the cross-validation procedure, we computed separate sets of pseudo-values in each of the training and test folds. For the same reason, we grew a new tree on every training fold.

### Calculation of the estimated survival probabilities

After having determined the optimal value of $m_{\text {stop}}(1)$, the gradient boosting models (with iteration numbers $m_{\text {stop}}(\mathcal {N})$) are successively fitted to the data in each node of the conditional inference tree. The last step of PRT is to calculate the individual-specific survival probabilities at all time points $t_1,\ldots , t_K$. This is done as follows: First, each observation $\tilde{\imath } \in \{1, \ldots , n \cdot K\}$ with covariate values $X_{\tilde{\imath }0}, X_{\tilde{\imath }1},\ldots , X_{\tilde{\imath }p}$ and time point $t_{\tilde{\imath }} \in \{t_1, \ldots , t_K \}$ is dropped down to a terminal node. Note that, by construction of the tree in Sect. 3.1, all *K* observations (= time points) referring to one individual are part of the same terminal node. Next, the fitted values $\{ \hat{f}_{\tilde{\imath }}^{[m_{\text {stop}}(\mathcal {N})]} \}_{\tilde{\imath }=1, \ldots , n\cdot K}$ are calculated from the boosting models in the terminal nodes. Finally, the estimated survival probabilities $\hat{S}_{\tilde{\imath }} (t_{\tilde{\imath }} | X_{\tilde{\imath }})$ are obtained by transforming the fitted values using the response function $h(\cdot )$, giving

12 $$\begin{aligned} \hat{S}_{\tilde{\imath }} (t_{\tilde{\imath }} | X_{\tilde{\imath }})= & {} \sum _{\mathcal {N} \in \mathcal {N}^{\mathcal {T}}} \mathbbm {1}_{\{ \tilde{\imath } \in \mathcal {N} \}} \, \cdot \, h \Big ( \hat{f}_{\tilde{\imath }}^{[m_{\text {stop}}(\mathcal {N})]} \Big ) \nonumber \\= & {} \sum _{\mathcal {N} \in \mathcal {N}^{\mathcal {T}}} \mathbbm {1}_{\{ \tilde{\imath } \in \mathcal {N} \}} \, \cdot \, \left( 1 - \exp \Big (- \exp \Big (- \hat{f}_{\tilde{\imath }}^{[m_{\text {stop}}(\mathcal {N})]}\Big ) \Big ) \right) \,, \ \ \ \tilde{\imath } = 1, \ldots , n \cdot K \,, \end{aligned}$$

where $\mathcal {N}^{\mathcal {T}}$ indicates the set of terminal nodes. Note that the above procedure also applies to any set of new individuals (possibly not contained in the available data), see Section S2 in the supplementary material for an illustration.

We emphasize that the node membership (indicated by $\mathbbm {1}_{\{ \tilde{\imath } \in \mathcal {N} \}}$) is determined by a set of binary decision rules depending on at most *D* split variables. Thus, the fitted survival probabilities in (12) contain (at most) *D*-way interactions between the split variables and each of the covariates selected by the boosting algorithm. Moreover, the multiplication of $\mathbbm {1}_{\{ \tilde{\imath } \in \mathcal {N} \}}$ with the baseline risk (contained in $\hat{f}_{\tilde{\imath }}^{[m_{\text {stop}}(\mathcal {N})]}$, see Equation (7)), defines a time-dependent effect in each terminal node. Of note, this effect does not only depend on the split variables but also on the covariates selected by higher-level boosting models (which are incorporated in the offset value $\hat{f}^{[0]}_{\tilde{\imath }}$).

**Fig. 2:**
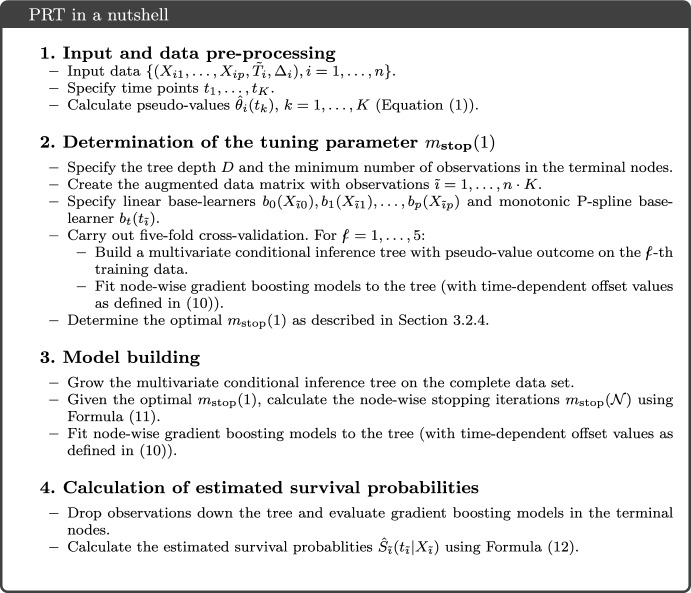
Schematic overview of the PRT method

Figure 2 presents a schematic overview of the PRT method, summarizing Sects. 3.1 to 3.3.

## Experiments

To investigate the properties of the PRT method, we carried out two simulation studies. In the first study (Sect. 4.1), the data-generating process was characterized by a tree with lognormal survival models in the terminal nodes (reflecting the true structure of a PRT model). The aim of this study was to analyze whether PRT was able to identify relevant covariates and subgroups defined by the interaction effects. In the second study (Sect. 4.2), the data-generating process was based on a lognormal model with an additive mixture of main and interaction effects. Here, the aim was to analyze the performance of PRT in the presence of model misspecification (since the additive model did not have a tree structure). Both studies were based on 100 Monte Carlo replications and $K=5$ time points (following the suggestions by Andersen and Pohar 2010). In each replication, we generated a training data set for model building and a test data set for model evaluation. The sample sizes of all data sets were set to $n=1000$. The time points were chosen as the mean empirical $10\%$, $30\%$, $50\%$, $70\%$, and $90\%$ quantiles of $\tilde{T_i}, i = 1, \ldots , 1000$, computed from 1000 additional individuals (generated independently using the data-generating processes of the simulation studies). Five-fold cross-validation on the training data was used to optimize the stopping parameter $m_{\text {stop}}(1)$.

Accuracy was measured by applying the fitted models to the test data set. We computed the mean squared error (MSE) defined by

13 $$\begin{aligned} \text {MSE} \,=\, \frac{1}{n\cdot K} \,\sum _{\tilde{\imath }=1}^{n\cdot K} \left( \hat{S}_{\tilde{\imath }}(t_{\tilde{\imath }}| X_{\tilde{\imath }}) - S_{\tilde{\imath }}(t_{\tilde{\imath }}| X_{\tilde{\imath }})\right) ^2 \, , \end{aligned}$$

where $\hat{S}_{\tilde{\imath }}(t_{\tilde{\imath }}| X_{\tilde{\imath }})$ and $S_{\tilde{\imath }}(t_{\tilde{\imath }}| X_{\tilde{\imath }})$ denote the estimated and the true survival probabilities, respectively, of observation $\tilde{\imath }$ at time $t_{\tilde{\imath }}$. To evaluate the error on the scale of the survival probabilities, we further computed the root mean squared error (RMSE), defined as the square root of (13). Additionally, we calculated the bias of the estimated survival probabilities (defined as the average deviation of the estimated survival probabilities from their respective true survival probabilities) and the Brier score (Kvamme and Borgan 2023). Discrimination ability was measured using the concordance index (*C*-index, Gerds et al. 2013). Bias and Brier score values were averaged across the five time points. The time horizon for the *C*-index was set equal to the largest time point $t_5$.

### Simulation study 1

We considered a model with ten covariates $x = (x_1, \ldots , x_{10})^T$ that followed a multivariate standard uniform distribution. The correlation matrix of this distribution was generated randomly and was the same in each replication (see Section S4 in the supplementary material). We used the method by Demirtas (2004) to sample the covariate values, restricting the pairwise Pearson correlations between the covariates to 0.5 in absolute value.

**Fig. 3:**
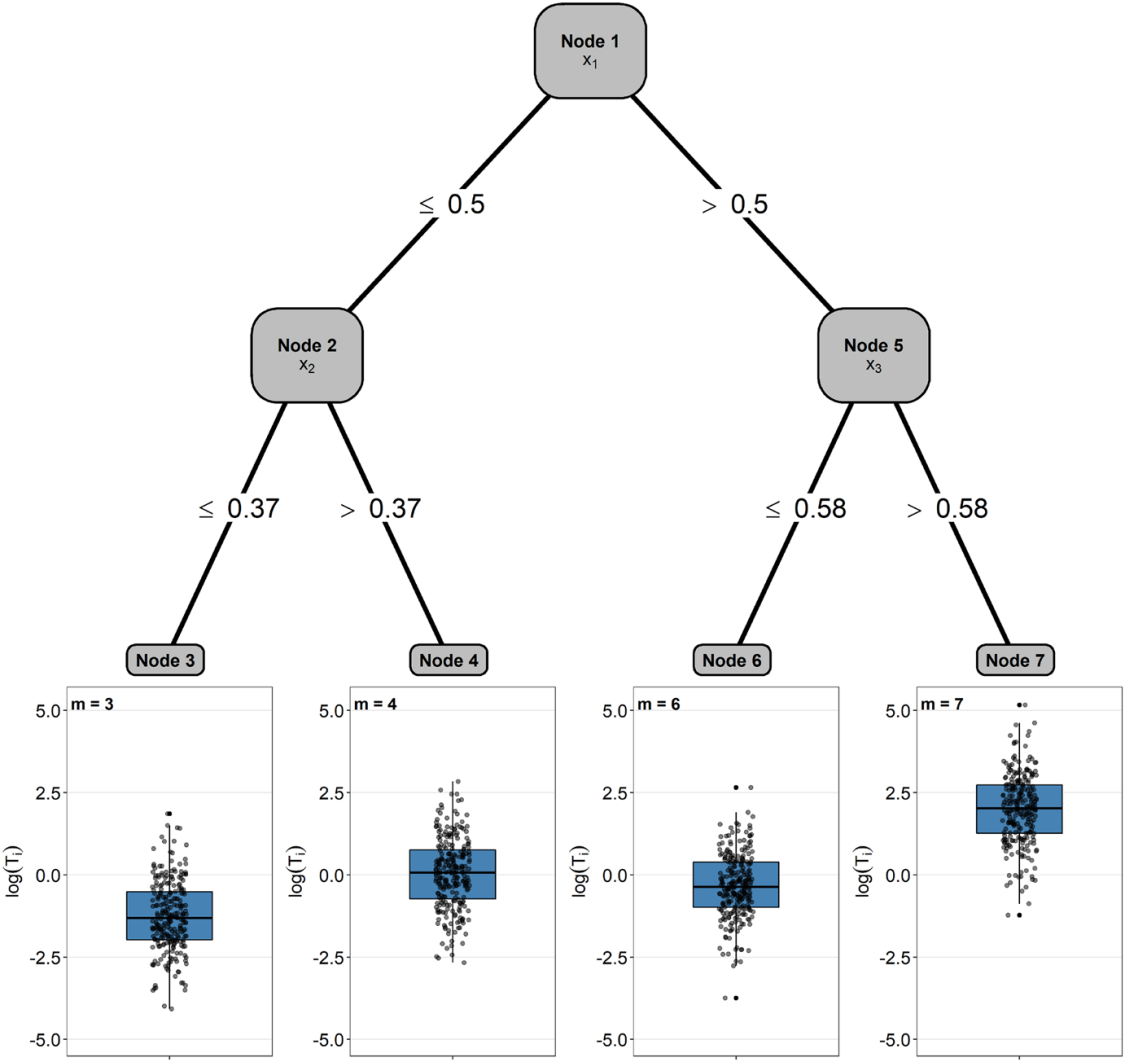
The plot illustrates the data-generating process of the first simulation study. The boxplots below the terminal nodes were generated from a random sample of size $n=1000$. They present the distributions of the survival times on the log scale

Imitating the structure of a tree with $D=2$, we first formed two subgroups of individuals defined by the decision rule $x_1 \le \xi _1$ vs. $x_1 > \xi _1$ with $\xi _1 = \text {median}(x_1)$. Afterwards, the groups of individuals with $x_1 \le \xi _1$ and $x_1 > \xi _1$ were split according to the decision rules $x_2 \le \xi _2$ vs. $x_2 > \xi _2$ and $x_3 \le \xi _3$ vs. $x_3 > \xi _3$, with $\xi _2 = \text {median}(x_2 \, | \, x_1 \le \xi _1)$ and $\xi _3 = \text {median}(x_3\,| \, x_1 > \xi _1)$, respectively (see Fig. 3). This resulted in four terminal nodes (indicated by the node numbers $m= 3,4,6,7$ in Fig. 3). In each of the terminal nodes, we generated lognormal survival times using different sets of informative and non-informative covariates. Denoting the node-wise linear predictors by

14 $$\begin{aligned} \eta _{im}&= \sum _{j = 1}^{10} \gamma ^{(m)}_{j} X_{ij} \, , \ \ \ i = 1, \ldots , n, \ \ \ m \in \lbrace {3,4,6,7 \rbrace } \, , \end{aligned}$$

the models in the terminal nodes were defined by

15 $$\begin{aligned} \log (T_i)= & {} \mathbbm {1}_{\lbrace {X_{i1} \le \xi _1 \rbrace }} \cdot \mathbbm {1}_{\lbrace {X_{i2} \le \xi _2 \rbrace }} \cdot \eta _{i3} \, +\, \nonumber \\{} & {} \mathbbm {1}_{\lbrace {X_{i1} \le \xi _1 \rbrace }} \cdot \mathbbm {1}_{\lbrace {X_{i2}> \xi _2 \rbrace }} \cdot \eta _{i4} \, +\, \nonumber \\{} & {} \mathbbm {1}_{\lbrace {X_{i1}> \xi _1 \rbrace }} \cdot \mathbbm {1}_{\lbrace {X_{i3} \le \xi _3 \rbrace }} \cdot \eta _{i6} \, +\, \nonumber \\{} & {} \mathbbm {1}_{\lbrace {X_{i1}> \xi _1 \rbrace }} \cdot \mathbbm {1}_{\lbrace {X_{i3} > \xi _3 \rbrace }} \cdot \eta _{i7} \, +\, \epsilon _i \,, \end{aligned}$$

with $\epsilon _i \sim N(0,1)$, $i=1\ldots , n$. In each terminal node, we set the coefficients $\gamma ^{(m)}_{j}$ of five randomly selected covariates to zero. The other coefficients were sampled from continuous uniform distributions with supports $[-1.25, 1.25]$ ($m=3$), $[-1, 1]$ ($m=4$), $[-1, 1]$ ($m=6$), and [0, 1] ($m=7$), see Table 2. Note that the values of the coefficients were the same in each replication. The censoring times $C_i$ were generated independently by the same process, resulting in a censoring rate of $50\%$.

**Table 2:** Covariate effects $\gamma ^{(m)}_j$, $j = 1, \ldots , 10$, in the first simulation study. The numbers $m \in \lbrace {3,4,6,7\rbrace }$ indicate the terminal nodes of the tree in Fig. 3

*m*	$\gamma ^{(m)}_1$	$\gamma ^{(m)}_2$	$\gamma ^{(m)}_3$	$\gamma ^{(m)}_4$	$\gamma ^{(m)}_5$	$\gamma ^{(m)}_6$	$\gamma ^{(m)}_7$	$\gamma ^{(m)}_8$	$\gamma ^{(m)}_9$	$\gamma ^{(m)}_{10}$
3	0	− 1.19	− 0.94	− 0.82	− 0.94	0	− 0.95	0	0	0
4	− 0.63	− 0.47	− 0.43	0.67	0	− 0.54	0	0	0	0
6	0	− 0.47	− 0.39	0	− 0.64	0	0	− 0.09	0	0.01
7	0.50	0.48	0	0.24	0	0	0.68	0.77	0	0

**Fig. 4:**
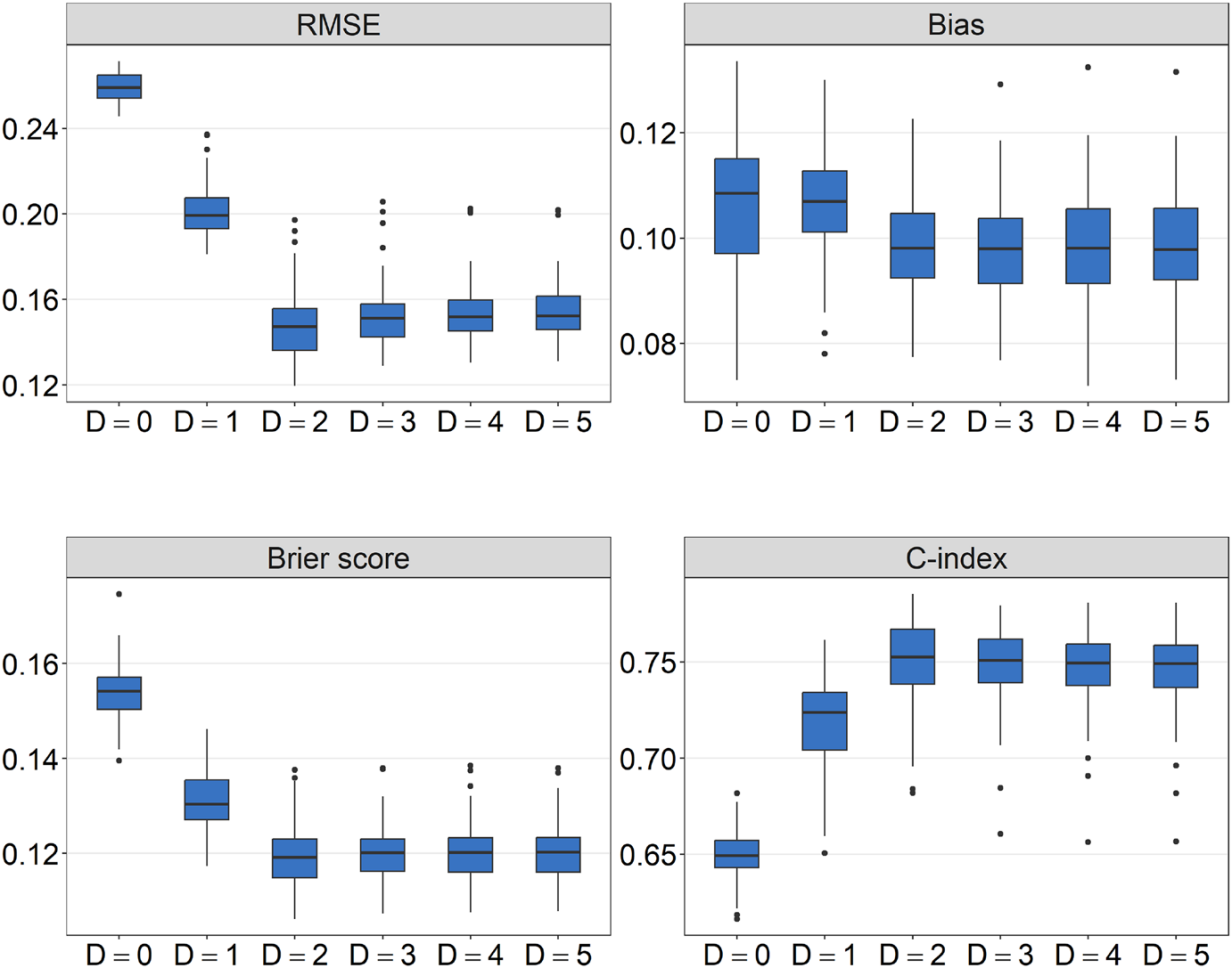
Results of the first simulation study. The boxplots present the RMSE, bias, Brier score, and *C*-index values that were obtained by applying the PRT method with varying tree depths ($D \in \{0,1,2,3,4,5\}$) to the training data and by evaluating the resulting model fits on the test data. Note that $D=2$ corresponds to the true tree depth, as defined by the data-generating process

We first investigated the effect of the tree depth on the performance of PRT. To this purpose, we evaluated the RMSE, the bias, the Brier score and the *C*-index on a grid of tree depths $D \in \lbrace {0,1,2,3,4,5 \rbrace }$. As expected, the absolute bias of PRT decreased with increasing *D* and remained nearly constant for $D > 2$ (see Fig. 4). The smallest RMSE, the smallest Brier score and the highest *C*-index values were obtained from the PRT model with $D=2$, matching the true depth defined by the data-generating process. Figure 4 also shows that the RMSE and Brier score values strongly increased and the *C*-index values strongly decreased when the tree depth was chosen too low. Specifically, the highest RMSE, the highest Brier score and the lowest *C*-index values were obtained from the PRT model with $D = 0$, which corresponds to a component-wise gradient boosting algorithm in the root node. These results demonstrate the negative effects obtained by ignoring relevant interactions between the covariates. By contrast, increasing *D* beyond the true depth $D> 2$ did not prove to be particularly harmful with regard to the RMSE, the Brier score and the *C*-index. Note, however, that large values of *D* tend to have a negative effect on the interpretability of PRT, increasing both the interaction depth and the number of terminal nodes (see Sect. 3.1.2).

**Fig. 5:**
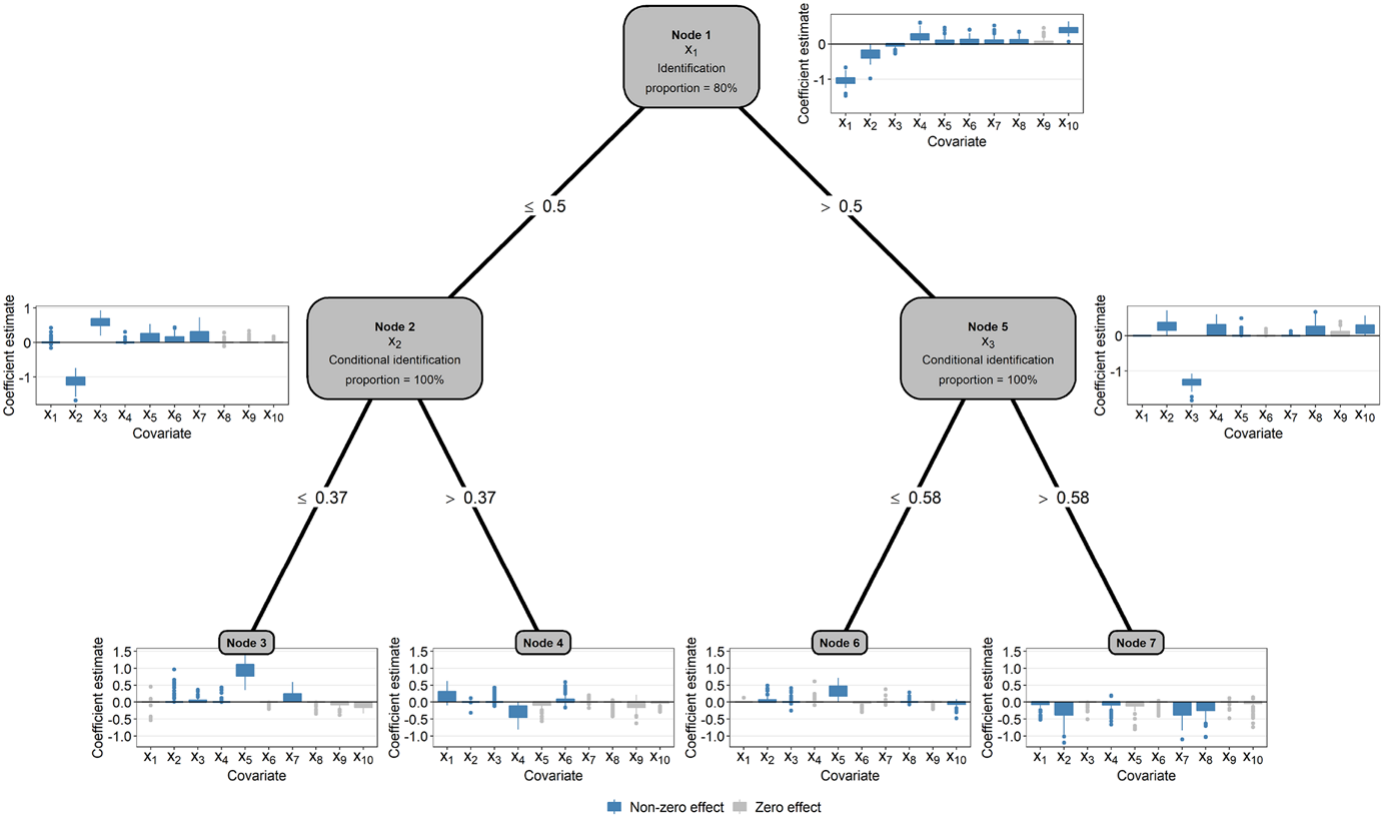
Results of the first simulation study ($D=2$, 100 Monte Carlo replications). The plot presents the percentages of correctly identified split variables as well as boxplots of the coefficient estimates obtained from the node-wise boosting fits. In Nodes 2–7, the percentages and coefficient estimates are conditional on having identified the split variable in the parent node. Blue and gray boxplots refer to informative covariates (defined by a non-zero effect in the present or in any of the lower-level nodes) and non-informative covariates, respectively. Coefficient estimates are zero if the respective base-learners were not selected by the gradient boosting algorithm

Second, we investigated the ability of PRT to identify the split variables and the informative covariates in the node-wise boosting models. To this purpose, we summarized the selection rates and the coefficient estimates of the PRT fits, setting $D=2$. As seen from the node labels in Fig. 5, the PRT fits identified the true underlying tree (first split at $x_1$ in the root node, second splits at $x_2$ and $x_3$ in Nodes 2 and 5, respectively) in $80\%$ of the Monte Carlo replications. By contrast, $2\%$ of the fits did only use two of the covariates $x_1, x_2, x_3$ for splitting; $18\%$ of the fits used the split variables $x_1, x_2, x_3$ but did not identify the correct order.

Regarding the coefficient estimates of the node-wise boosting models, we observed that informative covariates (defined by $\gamma ^{(m)}_j \ne 0$ in the present or in any of the lower-level nodes) were preferably selected and had higher coefficient estimates in absolute value than non-informative covariates. For example, $x_9$ (which did not have an effect on survival in any of the terminal nodes, Table 2) had a mean coefficient estimate of only 0.05 (upper right gray boxplot in Fig. 5). An important characteristic of PRT can be observed when comparing the estimated coefficients at various levels of the tree: Obviously, as the tree depth increased, the coefficient estimates of informative covariates became smaller in absolute value. This result is due to the time-dependent offset values in the boosting fits, which, by construction of the PRT method, capture the information of the model fits in higher-level nodes. Consider, for instance, the boosting fits in terminal Node 3 (lower left panel in Fig. 5): Although the coefficient estimates of the informative covariate $x_2$ appear to be small in this node, the strong negative effect of $x_2$ is clearly captured by the boosting fits in the parent node (see the blue boxplot to the left of Node 2). The respective coefficient estimates are passed to the daughter nodes via the time-dependent offset values, implying that they are also included in the boosting fits in terminal Node 3. This example demonstrates how the PRT method successively refines the coefficient estimates, passing relevant information from higher levels to the baseline risk in lower-level nodes.

### Simulation study 2

In the second simulation study, we considered a model with an interaction structure that did not match the tree structure of the PRT model. The aim of this study was to evaluate the accuracy of the survival probability estimates in the presence of model misspecification. Furthermore, we compared the PRT method to several alternative modeling techniques.

We considered a model with 30 covariates $x = (x_1, \ldots , x_{30})^\top$ that followed a multivariate normal distribution with mean zero and a randomly generated covariance matrix (see Section S4 in the supplementary material). All covariates had unit variance. For the main covariate effects we defined the linear predictor

16 $$\begin{aligned} \eta _{i,1} = \sum _{j=1}^{30} \gamma _j X_{ij} \,, \ \ \ i = 1, \ldots , n. \end{aligned}$$

Five out of the 30 covariates were informative, having non-zero coefficients $\gamma _j$. Furthermore, we considered all two-way interactions $x_j \cdot x_l$, $j\ne l$, $1 \le j <l \le 30$, and defined an interaction-only predictor by

17 $$\begin{aligned} \eta _{i,2} = \sum _{1 \le j < l \le 30} \gamma _{jl} X_{ij} X_{il} \,, \ \ \ i = 1, \ldots , n. \end{aligned}$$

The coefficients $\gamma _{jl}$ were set to zero if at least one of the covariates $x_j$ or $x_l$ was non-informative. All non-zero coefficients $\gamma _j$, $\gamma _{jl}$ were sampled from a continuous uniform distribution with support $[-1,1]$. They remained the same in each Monte Carlo replication. The combined predictor (including both the main effects and the interaction effects) was defined by

18 $$\begin{aligned} \eta _i&= \lambda \cdot \frac{\eta _{i,1} - \text {mean} (\eta _{i,1})}{\text {sd}{(\eta _{i,1})}} + (1 - \lambda ) \cdot \frac{\eta _{i,2} - \text {mean} (\eta _{i,2})}{\text {sd}{(\eta _{i,2})}} \, , \ \ \ i=1,\ldots , n\,, \end{aligned}$$

where $\text {mean} (\cdot )$ and $\text {sd} (\cdot )$ denote the empirical mean and standard deviation, respectively, and $\lambda \in [0,1]$ is a weighting factor that was included in (18) to analyze the impact of different weightings of the main and interaction effects on the performance of the PRT method. By definition, the predictor in (18) contained only main effects if $\lambda = 1$. Decreasing the value of $\lambda$ put more weight on the interaction terms, resulting in an interaction-only model if $\lambda = 0$. In our simulation study, we considered $\lambda \in \{ 0, 0.25, 0.5, 0.75, 1 \}$. Finally, we generated the survival times from a lognormal model defined by

19 $$\begin{aligned} \log (T_i)&= \frac{\eta _{i} - \text {mean} (\eta _i)}{\text {sd} (\eta _i)} + \epsilon _i \, , \ \ \ \epsilon _i \sim N(0,1)\,, \ \ \ i = 1, \ldots , n. \end{aligned}$$

The censoring times were generated in the same way as in the first simulation study, resulting in a censoring rate of $50\%$.

In addition to analyzing the performance of PRT, we compared our method to the following alternative approaches: (i) a regression tree built using model-based recursive partitioning (MOB, Zeileis et al. 2008, *MOB*), (ii) $L_2$ boosting with a pseudo-value outcome and tree base-learners of depth two (Friedman 2001, *BoostedTree*), (iii) a survival random forest (not relying on pseudo-values but on the untransformed data $(\tilde{T_i}, \Delta _i, X_i^\top )$, Ishwaran et al. 2008, *SRF*), (iv) a multivariate conditional inference tree without node-wise gradient boosting (built in the same way as in Sect. 3.1 using the multivariate pseudo-value outcome, *TreeOnly*), (v) component-wise gradient boosting with pseudo-value outcome (fitted to the data in the root node only, in the same way as in Sect. 3.2.1, *BoostingOnly*), (vi) the standard GEE approach with complementary log-log link and main effects only (cf. Sect. 2.1, *GEE*), (vii) an inverse-probability-of-censoring-(IPC)-weighted least squares model using log-transformed event times (with main effects only, Molinaro et al. 2004, *IPCW-LS*), (viii) a Cox proportional hazards model with main effects only (*Cox*), (ix) a parametric accelerated failure time model (based on log-transformed survival times and assuming normally distributed errors, *Lognormal*) and (x) the Kaplan-Meier estimator (*KaplanMeier*). For *MOB* and *TreeOnly* we used the same tree depths and minimum numbers of observations in the terminal nodes as for PRT. Along the same lines, we fitted main-effects Weibull models (not using pseudo-values but the untransformed data $(\tilde{T_i}, \Delta _i, X_i^\top )$) in the terminal nodes of the *MOB* tree. These models were based on the proportional hazards assumption, analogous to the complementary log-log link used in PRT. Note that the *BoostedTree* method did not include a time base-learner, effectively ignoring the dependency between pseudo-values of the same individual. Further note that the lognormal model (*Lognormal*) did not include any main and/or interaction terms with zero coefficients. Consequently, the structure of the lognormal model matched the definition of the data-generating process, and we expected this model to be superior to all other methods, providing lower reference values for the RMSE and the Brier score and an upper reference value for the *C*-index. In contrast, the KM method served as a covariate-free “null” model providing upper reference values for the RMSE and the Brier score and a lower reference value for the *C*-index. Further details on the specification and tuning of the methods are given in Section S4 in the supplementary material.

**Fig. 6:**
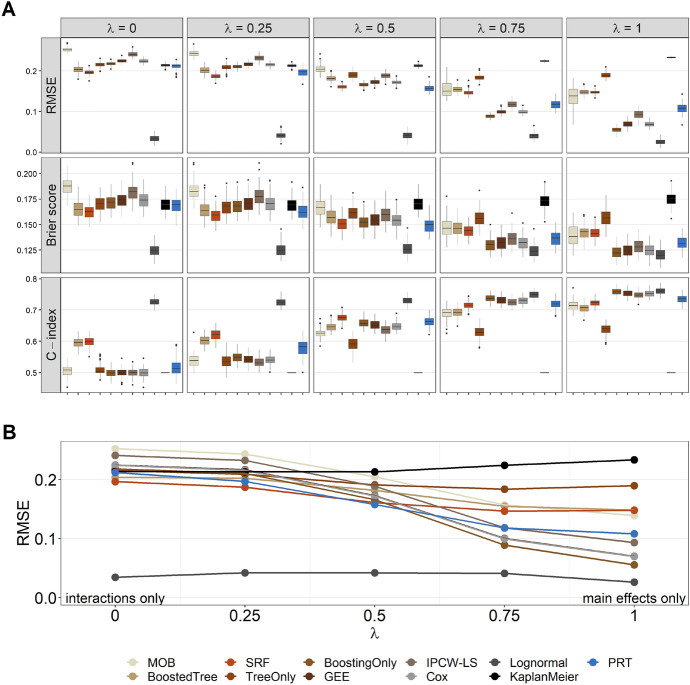
Results of the second simulation study ($D=2$, 100 Monte Carlo replications). **A** Boxplots of the RMSE, Brier score and *C*-index values, as obtained by evaluating the model fits on the 100 test data sets. **B** Mean RMSE values (across the replications). Note that *MOB* did not converge in some of the replications (failure rates = $2\%,1\%, 2\%,0\%$, and $1\%$ for $\lambda = 0, 0.25, 0.5, 0.75$, and $1$, respectively). The results of these models were excluded from the plots

The RMSE, Brier score and *C*-index values obtained from the second simulation study are presented in Fig. 6. Note that Fig. 6 includes the results for $D=2$ only, as this tree depth reflects the two-way interaction effects in (17). The results for $D>2$ were very similar (see Section S5 in the supplementary material). Estimates of the bias of PRT (mostly ranging between $-0.05$ and 0.05 for all tree depths) are presented in Section S6 in the supplementary material.

As expected, the *Lognormal* method outperformed the other methods in all settings, resulting in the smallest RMSE values, lowest Brier score values, and highest *C*-index values by far. As described above, this was because the *Lognormal* model matched the structure of the true data-generating process. For $\lambda = 0$ (no main effects, two-way interactions only), PRT outperformed all other methods except *SRF*, *BoostedTree* and *Lognormal*. Again, this is a plausible result, as the *BoostedTree* algorithm was defined by tree base-learners of depth two, resulting in an additive combination of two-way interactions (thereby matching the true structure of the predictor in case $\lambda = 0$). Similarly, *SRF* is a tree ensemble that is expected to outperform single-tree methods like PRT in terms of prediction accuracy. The *MOB* approach resulted in rather high RMSE values, high Brier score values and low *C*-index values, which was likely due to the high variability of the Weibull fits in the terminal nodes. Note that, unlike PRT, *MOB* does not perform variable selection in the terminal nodes and is not stabilized by offset values containing information from higher-level nodes. This increases the variability of coefficient estimates when the number of covariates is large relative to the node size. For the same reason, *MOB* could not even be fitted in some of the Monte Carlo replications, see the caption of Fig. 6. We also observed that the simple Kaplan-Meier estimator (serving as a covariate-free null model) performed quite well for $\lambda = 0$. This result might be explained by the large numbers of zero main effects (30 out of 30 when $\lambda =0$) and zero interaction terms (425 out of 435), making it hard for any modeling technique to approximate the true model structure.

When increasing the value of $\lambda$ to 0.25 (corresponding to models with non-zero main effects but $25\%$ weight on the interaction terms), the PRT method performed better in terms of RMSE, Brier score and *C*-index than all other methods (except *SRF* and *Lognormal*, see above). When main effects and interactions were weighted equally ($\lambda = 0.5$), the PRT method performed best with respect to both RMSE and Brier score (except *Lognormal*, as expected). *C*-index values were highest for *Lognormal* (as expected), followed by *SRF* and PRT with only minor differences between the latter two. Note, in particular, that PRT performed better than *TreeOnly* and *BoostingOnly* in the scenarios with $\lambda \le 0.5$. This result clearly demonstrates the benefit of combining the two methods if both main and (relevant) interaction effects are present.

In the scenarios with $\lambda \in \{0.75, 1\}$, all tree-based approaches (PRT, *MOB*, *BoostedTree*, *SRF*, *TreeOnly*) were outperformed by the main-effects models *BoostingOnly*, *GEE*, *IPCW-LS*, *Cox* and *Lognormal*. This is another plausible result, as the data-generating process either put a small weight on the interaction terms ($\lambda = 0.75$) or completely excluded the interaction terms ($\lambda = 1$) in these scenarios. Among the main-effects models, *BoostingOnly* performed best (except *Lognormal*, see above), demonstrating the benefit of variable selection and shrinkage in scenarios with a larger number of non-informative covariates. The standard approaches (*GEE*, *Cox*) also resulted in RMSE values that were substantially smaller than those of the tree-based methods. On the other hand, we note that PRT performed best among the tree-based methods, coming closest to the RMSE values of the main-effects models when $\lambda \in \{0.75, 1\}$. Of note, among the single-tree methods (PRT, *MOB* and *TreeOnly*), PRT was the only method that was able to outperform the ensemble method *SRF* in these settings (with respect to all considered performance measures).

## Application

To illustrate the PRT method, we analyzed data from the SUCCESS-A trial (NCT02181101), which was a multicenter randomized phase III study that enrolled 3,754 patients with a primary invasive breast cancer between September 2005 and March 2007. All patients had a high recurrence risk, meaning that the SUCCESS-A study population did not constitute a random sample from the general population; for details on the inclusion/exclusion criteria and the design of the study see de Gregorio et al. (2020). The study had two treatment arms, with patients either receiving one of the standard chemotherapy regimens (control group) or standard chemotherapy with the addition of gemcitabine (experimental group). The randomization ratio was 1:1. The aims of SUCCESS-A were to compare the two groups with respect to disease-free survival (DFS) and overall survival (OS) within a five-year follow-up period. Here we focus on DFS, which, according to the STEEP system, was defined as the period from the date of randomization to the earliest date of disease progression (distant metastases, local and contralocal recurrence, and secondary primary tumors or death from any cause, de Gregorio et al. 2020). Since the definition of DFS included death from any cause, we did not consider death as a competing event.

Patients were censored at the last date on which they were known to be disease-free, resulting in an event rate of $12.2\%$ (458 events in 3,754 patients). The maximum observation time was 5.5 years (6 months of chemotherapy followed by 5 years of follow-up; median 5.2 years, first quartile 3.7 years, third quartile 5.5 years). In addition to the survival times, the study collected data on several established prognostic factors, including age at randomization (*age*, measured in years), body mass index (*BMI*, measured in $kg/m^2$), tumor stage (*stage*, four categories, pT1/pT2/pT3/pT4), tumor grade (*grade*, three categories, G1/G2/G3), lymph node status (*nodal status*, two categories, pN0/pN+), tumor type (*type*, three categories, ductal/lobular/other), menopausal status (*meno*, two categories, pre-/post-menopausal), and receptor status for estrogen (*ER*), progesterone (*PR*), and *HER2* (two categories each, negative/positive), see de Gregorio et al. (2020). A descriptive summary of the prognostic variables is given in Table S3 in Section S7 in the supplementary material.

A key issue in the development of treatment guidelines for breast cancer is the identification of patient subgroups with possibly different risks of disease progression (Coates et al. 2015; Senkus et al. 2015). To illustrate the PRT method, we investigated the existence of such subgroups in the SUCCESS-A data, noting that tree-based methods have a long tradition in medical risk assessment (including, among other techniques, univariate survival trees, LeBlanc and Crowley 1992; Bacchetti and Segal 1995, tree-structured classification and regression, Ciampi et al. 1995; Puth et al. 2020, and mixtures of survival trees, Jia et al. 2022). In addition to using the aforementioned prognostic factors as covariates, we included the group status (*group*, two categories, control/experimental) and *time* (monotonic P-spline base-learner) in our model. Pseudo-values for DFS were computed at 1, 2, 3, 4, and 5 years ($K=5$). The depth of the regression tree was fixed at $D=3$. Our rationale for choosing this number was that it allowed for capturing interaction effects while, at the same time, resulting in a tree with a reasonably simple interpretation (analogous to the specification of the interaction order in linear regression). Patients with missing values in any of the variables (102 patients, $2.7\%$) were excluded from analysis, resulting in an analysis data set with *n* = 3,652 patients. The accuracy of the PRT model was evaluated by computing five-fold cross-validated values of the concordance index (*C*-index, Uno et al. 2011, with five-year time horizon) and the Brier score (Kvamme and Borgan 2023, averaged across the five time points).

As mentioned above, the aim of applying PRT to the SUCCESS-A data was to illustrate our method but not to optimize it with respect to prediction accuracy. For sensitivity analysis, we additionally present the Brier score and *C*-index values obtained from PRT with tree depths fixed at $D \in \lbrace {2,4,5\rbrace }$, corresponding to maximum numbers of 4, 16 and 32 terminal nodes, respectively. We also compared PRT to the methods described in Sect. 4.2. Note that we had to fix the tree depth of the *MOB* method at $D=1$, as larger values resulted in convergence issues.

**Fig. 7:**
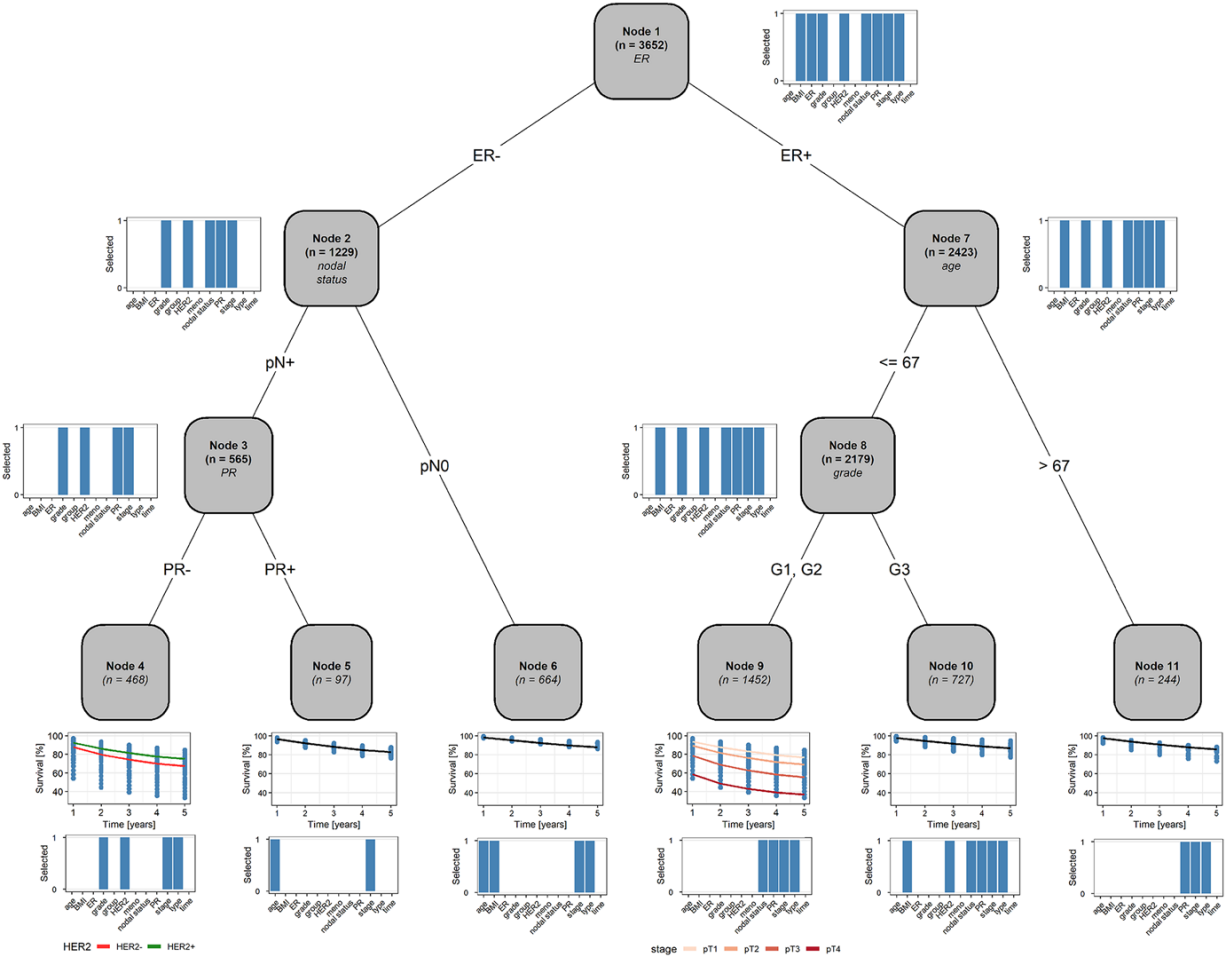
Analysis of disease-free survival in the SUCCESS-A study data. The figure presents the results obtained from fitting a PRT model with $D=3$, showing the selected split variables and the sizes of the patient subgroups in the nodes. The blue bars refer to the base-learners selected in the node-wise boosting models. The blue dots and the black lines refer to the fitted values and their averages in the terminal nodes. In Node 4, the mean estimated DFS function of the group of “triple negative” patients (i.e. negative *ER*, *PR* and *HER2*, von Minckwitz et al. 2012) is marked red. The green line refers to mean estimated DFS in the group of HER2 receptor-positive patients. The colored lines in Node 9 refer to the mean estimated DFS functions stratified by tumor stage (light red = pT1, dark red = pT4)

The regression tree obtained from the PRT fit is shown in Fig. 7. Overall, the tree structure reflected several established prognostic factors and subgroups, which have been frequently reported in the literature and have also been included in treatment guidelines for breast cancer (Coates et al. 2015; Senkus et al. 2015). Specifically, the first split variable (selected in the root node) was *ER*, indicating the importance of this variable in adjuvant hormonal and chemotherapeutic treatment regimens. The survival advantage of estrogen receptor-positive patients (Goldhirsch et al. 2003) is reflected by the estimated five-year DFS probabilities, which were $80.87\%$ on average in Nodes 4, 5, and 6 and $89.89\%$ on average in Nodes 9, 10, and 11 (corresponding to estrogen receptor-negative patients and estrogen receptor-positive patients, respectively). The split variables in the second level of the tree were *nodal status* and *age* (threshold = 67 years), reflecting the higher risk of lymph node-positive patients (Senkus et al. 2015) and the increased risk of patients aged 67 or older, respectively (Chen et al. 2016). This result is confirmed by the average estimated five-year DFS probabilities, which were smaller in lymph node-positive patients than in lymph node-negative patients ($72.46\%$, Nodes 4 and 5, vs. $88.04\%$, Node 6), and were higher in patients aged $\le 67$ years than in patients aged $> 67$ years ($90.34\%$, Nodes 9 and 10, vs. $85.82\%$, Node 11). Patients with negative estrogen receptor status and positive lymph node status were further split into subgroups defined by *PR*. Of note, progesterone receptor-negative patients were estimated to have the lowest average five-year DFS probabilities ($70.31\%$, Node 4). This group of patients also included the high-risk group of “triple negative” patients (negative *ER*, *PR*, and *HER2*, von Minckwitz et al. 2012), given through the base-learner for *HER2* selected by the boosting model in Node 4. In line with the literature (von Minckwitz et al. 2012), the subgroup of triple negative patients had lower estimated five-year DFS probabilities on average than HER2 receptor-positive patients in Node 4 (red vs. green lines in the lower left panel of Fig. 7). Another prognostic variable is *grade*, which was selected as split variable in the group of patients $\le 67$ years in Node 8. We note that the grouping of *grade* (G1/G2 vs. G3) reflects the grouping in current treatment guidelines for breast cancer (Coates et al. 2015; Senkus et al. 2015). As expected, patients with a low or intermediate grade had a higher estimated five-year DFS probability (G1/G2, $92.03\%$, Node 9) than patients with a high grade (G3, $86.97\%$, Node 10). Furthermore, tumor stage (selected by the boosting model) had a strong impact on survival in Node 9 (see colored lines in Fig. 7). Regarding the treatments investigated in the SUCCESS-A trial, we observed that the *group* was not selected in any of the nodes, neither for splitting nor as base-learner in the boosting models. This is in line with the findings in the original study report by de Gregorio et al. (2020), who concluded that the addition of gemcitabine to standard chemotherapy did not improve DFS.

The five-fold cross-validated Brier score and *C*-index values obtained from PRT and the alternative methods are shown in Table 3. It is seen that the Brier score and *C*-index values obtained from PRT were similar for all considered tree depths, suggesting that higher values of *D* did not increase predictive performance but only led to a more difficult interpretation of the models. Overall, PRT were very similar to the alternative methods in terms of prediction accuracy (except *TreeOnly* and *KaplanMeier*, which performed worse than PRT as expected, and *BoostedTree* and *IPCW-LS*, which also performed worse than PRT). These results support the plausibility of the above interpretations and the validity of the PRT model in Fig. 7.

**Table 3:** Analysis of the SUCCESS-A study data. The table presents the five-fold cross-validated values of the time-averaged Brier score and the *C*-index at 5 years, as obtained from fitting PRT (with $D \in \lbrace {2,3,4,5\rbrace })$ and the alternative methods to the study data

Method	Average Brier score	*C*-index at 5 years
PRT ($D=2$)	0.069	0.660
PRT ($D=3$)	0.069	0.660
PRT ($D=4$)	0.069	0.651
PRT ($D=5$)	0.069	0.662
*MOB*	0.069	0.666
*BoostedTree*	0.081	0.618
*SRF*	0.069	0.668
*TreeOnly* ($D=2$)	0.070	0.596
*TreeOnly* ($D=3$)	0.070	0.621
*TreeOnly* ($D=4$)	0.069	0.639
*TreeOnly* ($D=5$)	0.069	0.644
*BoostingOnly*	0.068	0.670
*GEE*	0.070	0.667
*IPCW-LS*	0.291	0.597
*Cox*	0.068	0.670
*Lognormal*	0.068	0.669
*KaplanMeier*	0.072	0.500

## Discussion

This paper presents a semi-parametric approach for building time-to-event models with a pseudo-value outcome. Our method, entitled pseudo-value regression trees (PRT), results in a piecewise regression model for the survival function, where the “pieces” are obtained by recursively partitioning the covariate space. As described in Sect. 3, developing a model tree algorithm for pseudo-values involved, among other components, a method for multivariate tree building, a loss function for non-normal continuous outcomes, and an appropriately defined time base-learner to ensure monotonicity of the probability estimates. Our numerical experiments in Sects. 4 and 5 demonstrated that the PRT method was able to identify relevant covariates and interactions (Sect. 4.1), showed a favorable estimation accuracy (Sect. 4.2), and yielded highly plausible results in our application on primary invasive breast cancer (Sect. 5). Importantly, by restricting the tree depth to a moderate value ($D \le 5$), the fitted PRT models had an easily accessible interpretation (see e.g. Fig. 7). This is considered to be a major advantage when the focus is not solely on prediction accuracy, especially when compared to black-box methods like support vector machines, random forests, or deep neural networks (see e.g. Mogensen and Gerds 2013; van der Ploeg et al. 2014; Zhao and Feng 2020; Rahman et al. 2021).

Conceptually, the PRT method belongs to the class of “direct” modeling approaches, relating the covariates directly to the survival probabilities instead of relating them “indirectly” to $S(t|X_i) = \exp (- \int _0^t \lambda (u|X_i) du )$ via the hazard function $\lambda (t|X_i)$ (as done e.g. by the Cox model and Aalen’s additive hazard model). Prominent examples of direct models are the proportional odds model and the Cox-Aalen model, which can be fitted to a set of censored time-to-event data using inverse-probability-of-censoring-(IPC)-weighted binomial regression (Scheike et al. 2008, see also Grøn and Gerds 2014 and the references therein). Analogous to pseudo-value regression, these models provide estimates of $S(t_k | X_i)$ on a pre-defined grid of time points $t_k = t_1, \ldots , t_K$. The same is true for the hierarchical modeling approach by Garcia et al. (2019), which is a mixture of binomial regression and pseudo-value regression; instead of using IPC weights (effectively excluding censored individuals from the estimation equation), the authors replaced the binary values of *censored* individuals by pseudo-values and fitted the (pseudo-)binomial model within the generalized additive modeling framework. Hothorn et al. (2014) proposed the class of *conditional transformation models*, which is a general approach to model the distribution function $F(t|X_i) = 1 - S(t|X_i)$ conditional on a set of covariates (including direct survival models as special cases). Of note, Hothorn et al. (2018) developed a likelihood-based approach for the modeling of $F(t|X_i)$ that does not require pre-specification of a grid of time points (see also Hothorn 2019 and the references therein). Similar to Garcia et al. (2019), Hothorn et al. (2018) proposed to model the baseline risk and the covariate effects using basis functions. Despite the flexibility of the aforementioned approaches, we emphasize that the *building* of survival models (in particular, the specification of the model structure) remains a challenging task. Tree-based methods like PRT are useful in addressing this issue, providing tools for variable selection and the identification of interaction effects. On the other hand, the selection steps performed by PRT (and also by related tree methods) preclude the application of standard hypothesis tests in the nodes (Loh et al. 2019). As a consequence, tree-based methods like PRT should be handled with care if the model structure is fixed and if statistical inference is of major interest.

The PRT approach is also related to other methods for building model trees. In Sect. 4, for instance, we used the *model-based recursive partitioning* approach (MOB) as a comparator to PRT. Conceptually, PRT and MOB are of similar nature; however, they differ with respect to their tree building approaches: While PRT uses the generalized correlation coefficient in (5) for (multivariate) recursive partitioning, MOB applies a test for parameter instability (Zeileis and Hornik 2007) to determine the split variable in each node. In contrast to (5) (which is based on the bivariate relationships between the pseudo-values and the covariates), this instability test requires the node-wise fitting of a regression model including all covariates. As a consequence, MOB is usually more sensitive in detecting interaction effects in the models of interest (controlling for possible confounding instead of considering the “marginal” distributions as in (5)); on the other hand, the validity of the test results might be compromised by multicollinearity, especially when the number of covariates is large relative to the node size (see Sect. 4). To the best of our knowledge, there exists no regularized version of the MOB algorithm (performing e.g. variable selection like the boosting models in PRT). We further note that the current implementation of MOB in the R package **partykit** does not allow for modeling correlated observations (e.g. via mixed-effects models or a multivariate tree as in PRT). Similar arguments hold for the GUIDE algorithm, which has recently been extended by Loh et al. (2019) to build model trees within the proportional hazards framework.

As stated in Sect. 1, the consistency results by Graw et al. (2009) rely on the assumption that the censoring times $C_i$ are independent of the survival times $T_i$ (“random censoring”). Under this assumption, it can be shown that $\text{ E }[\hat{\theta }_i (t_k) | X_i] \,{\mathop {\rightarrow }} \text{ E }[\mathbbm {1}_{\{ T_i > t_k\}} |X_i] = S(t_k | X_i)$, so that using the pseudo-values as outcome variable in a statistical model (as done by PRT) is equivalent to substituting the outcome values of interest (here, the unobserved survival probabilities) by consistent estimates of these values. Later, Binder et al. (2014) showed that the random censoring assumption can be relaxed to allow for censoring times $C_i$ that are only *conditionally* independent of $T_i$ given the covariate values $X_i$. More specifically, the authors considered a scenario where pseudo-values are based on the Aalen-Johansen estimator of the cumulative incidence function $\text{ P }(T_i \le t_k)$. By fitting a regression model for the censoring survival function $G(t_k|X_i):= \text{ P }(C_i > t_k|X_i)$ and incorporating the resulting IPC weights in the Aalen-Johansen estimator, they were able to eliminate the bias occurring from a “naive” calculation of the pseudo-values ignoring dependency on $X_i$. Analogously, the PRT method could be extended to scenarios with covariate-dependent censoring. This could be done by replacing the Kaplan-Meier estimators in (1) by one minus the respective IPC-weighted Aalen-Johansen estimators. We point out that the results by Binder et al. (2014) rely on the correct specification of the regression model for $G(t_k|X_i)$.

We further note that the pseudo-value methodology is not restricted to the estimation of survival probabilities from right-censored data. For example, Andersen and Pohar (2010) considered a general class of functionals of the form $\text{ E}\left[ \psi (T_i) \vert X_i \right]$, suggesting that any of these functionals could be estimated by an appropriately defined pseudo-value regression model. In the same manner, the PRT approach could be adapted to a wider class of functionals, an obvious example being the cumulative incidence function in competing-risks analysis. Following the idea described in Zhao et al. (2020), PRT could also be applied for dynamic risk prediction. Furthermore, PRT can easily be extended to incorporate left-truncated survival times referring to individuals not yet at risk at time $t=0$. Provided that the truncation times are independent of the survival times $T_i$ (at least conditional on $X_i$, see above), this could be done by an appropriate definition of the risk sets used in the calculation of the Kaplan-Meier estimators in (1). With regard to the latter, robustness can be increased by computing “stopped” pseudo-values $\hat{\theta }_{i}(t_k)$ that are based on only those individuals who entered the sample before the respective time points $t_k$. For details, see Grand et al. (2019).

Finally, we would like to note that PRT is, in general, applicable to high-dimensional scenarios with $p > n$. Naturally, very large numbers of covariates may result in increased run-times (which are mainly due to the permutation tests conducted for the selection of the split variables). It should also be noted that both conditional inference trees and gradient boosting usually require some sort of pre-filtering in order to work well in “ultra-high”-dimensional scenarios with $p \gg n$. The latter aspects are, of course, not specific to PRT but apply to many methods for modeling high-dimensional data (e.g. to penalized regression and even to random forests).

**Software**

All computations were carried out using the R Language for Statistical Computing (version 4.1.2, R Core Team 2022). An implementation of the PRT method is available at https://www.imbie.uni-bonn.de/cloud/index.php/s/5oZDBSJjW4pLjtb. Details are given in Section S1 in the supplementary material.

### Supplementary Information

Below is the link to the electronic supplementary material.

Supplementary file 1 (PDF 1045 KB)
