# Supplementary material for: Pseudo-value regression trees
Source: Lifetime Data Anal. 2024 Feb 25;30(2):439–71. doi: 10.1007/s10985-024-09618-x (PMC11297840; doi:10.1007/s10985-024-09618-x)
Supplement: Supplementary file 1 — (PDF 1045 KB) [file 10985_2024_9618_MOESM1_ESM.pdf]

Lifetime Data Analysis
https://doi.org/10.1007/s10985-024-09618-x

Supplementary Material to

Pseudo-value regression trees

Alina Schenk · Moritz Berger · Matthias Schmid

Received: 23 January 2023 / Accepted: 19 January 2024

S1 Implementation of pseudo-value regression trees in R

The main function of the PRT implementation is

```
PRT(data, covariates, cutoffs_pseudo, time_col,
status_col, id_col, D = 3, fold = 5, center = TRUE)
```

with the arguments given in Table S1. An examplary illustration of the PRT method is given in the **readme.html** file at `https://www.imbie.uni-bonn.de/cloud/index.php/s/5oZDBSJjW4pLjtb`.

Pseudo-values were computed using the `pseudosurv` function of the R package **pseudo** (version 1.4.3, Pohar Perme and Gerster 2017). For growing the multivariate conditional inference trees, we applied recursive partitioning as implemented in the `ctree` function of the R package **partykit** (version 1.2-20, Hothorn and Zeileis 2015). As a remark, note that there are many alternative options to build the tree (specified in the `ctree_control` option of `ctree`), including the application of a quadratic form for the calculation of the univariate test statistics. For details, we refer to Hothorn et al. (2006). The **partykit** package was also used to extract node and split information. Node-specific boosting models were fitted using the `gamboost` function of the R package **mboost** (version 2.9-8, Hothorn et al. 2022a). In order to implement the new loss function (see Equation (8) of the main paper), we set up a new `Family` called `GaussCloglog`. Table S2 presents the specification of the base-learners used in PRT (see Section 3.2 of the main paper).

Alina Schenk
Institute of Medical Biometry, Informatics and Epidemiology, Medical Faculty, University of Bonn
Tel.: +49 (0)228-287-10548
E-mail: schenk@imbie.uni-bonn.de
ORCID: 0000-0003-1998-2625

Moritz Berger
Institute of Medical Biometry, Informatics and Epidemiology, Medical Faculty, University of Bonn
ORCID: 0000-0002-0656-5286

Matthias Schmid
Institute of Medical Biometry, Informatics and Epidemiology, Medical Faculty, University of Bonn
ORCID: 0000-0002-0788-0317

Argument	Description
`data`	Data frame that the PRT method should be applied to.
`covariates`	Vector of covariate names that should be included in the analysis (used for tree building and for node-wise boosting). Covariate names should match the column names of `data`.
`cutoffs_pseudo`	Vector of length K containing the time points pseudo-values should be calculated for. The maximum time point should not exceed the maximum observed time in the data.
`time_col`	Name of the column that contains the observed times.
`status_col`	Name of the column that contains the event status.
`id_col`	Name of the column that contains the individual id.
`D`	Depth of the regression tree.
`fold`	Number of cross-validation folds to determine the optimal iteration number in the node-wise boosting models.
`center`	TRUE/FALSE indicating whether continuous covariates should be mean-centered.

Table S1 Arguments of the R function `PRT`.

Type of covariate	Base-learner	Specifications
x_0	`bols`	`intercept = FALSE`
Continuous covariates	`bols`	`intercept = FALSE`
Categorical covariates	`bols`	`intercept = FALSE`<br>coded as factor
Time<br>(as continuous variable)	`bmono`	`intercept = FALSE` and<br>`constraint = 'increasing'`

Table S2 Specification of the base-learners used in PRT (included as arguments of the `gamboost` function of the R package **mboost**, Hofner et al. 2014).

S2 Estimation of survival probabilities

Figure S1 visualizes the estimation of the survival probabilities of a new observation (not necessarily included in the training data set).

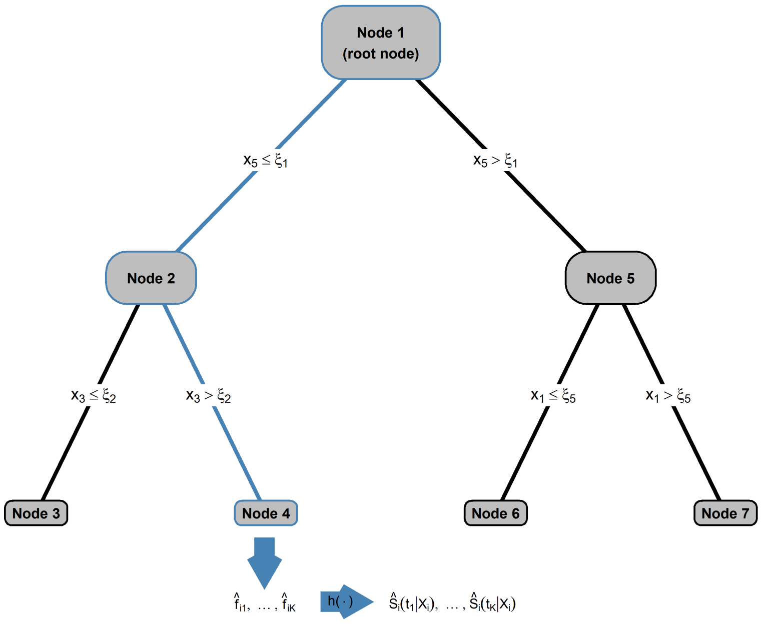

Fig. S1 Given a new observation $X_i = (X_{i0}, X_{i1}, \ldots, X_{ip}) = (1, X_{i1}, \ldots, X_{ip})$, the prediction of time-dependent survival probabilities is analogous to LMT: First, the new observation is dropped down the tree until a terminal node specifying the subgroup the individual falls into, is reached. For the exemplary regression tree given here, the path of the new observation from the root node to its specific terminal node is given as $(1 \rightarrow 2 \rightarrow 4)$ (marked blue). Having reached Node 4, the estimated survival probability $\hat{S}_i(t_k|X_i)$ at time t_k is calculated by evaluating the boosting model of this terminal node, obtaining the linear predictor $\hat{f}_{ik}$ and plugging it in the inverse complementary log-log function $h(\cdot)$.

S3 Details on time-dependent effects

To illustrate the ability of PRT to model time-dependent effects, we conducted a simulation study visualizing the time-dependent effect of a single covariate Z. In each of the 100 simulation runs, we generated survival times for $n = 1000$ independent individuals from a piecewise exponential model. The covariate values Z_i, $i = 1, \ldots, n$, were generated from a uniform distribution on $[0, 1]$. The piecewise hazard rates were set to

$$\lambda_i(t|Z_i) = \begin{cases} 1 & \text{if } t \leq 0.2 \ , \\ 2 \cdot Z_i & \text{if } t > 0.2. \end{cases} \tag{1}$$

By definition, the hazard rate for the i-th individual was constant and did not depend on Z_i up to time point $t = 0.2$. For time points $t > 0.2$, the hazard rate increased with the value of Z_i. As a consequence, the data-generating process included a time-dependent effect of Z_i on the hazard rate, and thus also on the survival function. Censoring times were generated from an exponential distribution whose rate parameter was adjusted such that the censoring rate was approximately 50%. Pseudo-values were computed at the five time points $(t_1, \ldots, t_5) = (0.1, 0.2, 0.3, 0.4, 0.5)$, and PRT were fitted to the data. The tree depth was set to $D = 1$ for illustrative purposes.

Figure S2 shows a summary of the time-dependent fitted values (on the predictor scale) that were obtained from the two daughter nodes after splitting in Z and fitting node-wise boosting models. The dots represent the averages of the fitted values (on the predictor scale, denoted by $\bar{\hat{f}}$). Averages were taken over all 100 simulation runs and all individuals in the respective nodes. Note that the threshold ξ for X_1 was slightly different in each simulation run. Also note that both time and Z were considered as base-learners in the boosting algorithm.

As seen from Figure S2, the mean fitted values in Node 3 (corresponding to large values of Z_i) were higher at all time points than the respective values in Node 2 (corresponding to small values of Z_i). This result is in line with the positive effect of Z on the hazard, which translates to a positive effect on $\bar{\hat{f}}$ (by definition of the complementary log-log link $g(x) = -\log(-\log(1 - x))$). For time points $t \leq 0.2$, the two lines in Figure S2 run almost in parallel. As expected, this result corresponds to the absence of a time-dependent effect of Z on the hazard for $t \leq 0.2$. More specifically, the vertical distance between the lines at $t = 0.1$ and $t = 0.2$ can be interpreted as the average difference in *main effects* of Z. For time points $t > 0.2$, the two lines no longer run in parallel. Instead, the slope of the line corresponding to Node 3 (including high values of Z_i) is higher than the respective slope in Node 2 (including low values of Z_i). Again, this result is in line with the above definition of the piecewise hazard, which incorporates a positive effect of Z for $t > 0.2$ – and thus also implies an increased slope of the fitted values in Node 3. Taken together, PRT were able to capture the time-dependent effect of Z on survival.

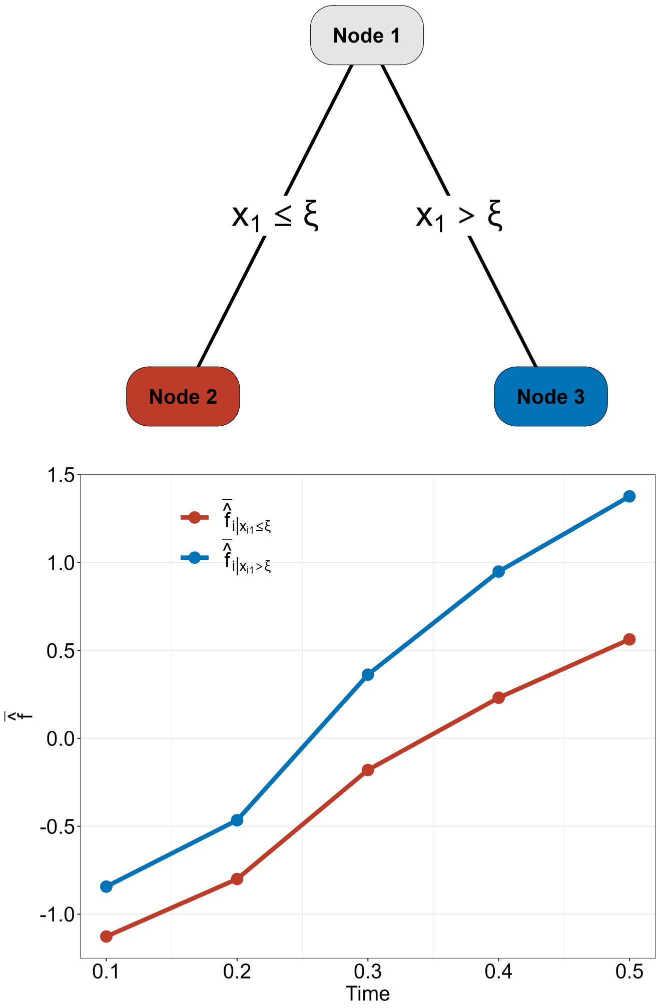

Fig. S2 Results obtained from fitting PRT to piecewise exponential survival data.

S4 Details on the simulations

Simulation Study 1

For the first simulation study, values from a multivariate uniform distribution were drawn using the `draw.d.variate.uniform` function of the R package **MultiRNG** (version 1.2.4, Demirtas et al. 2021). This package implements the method by Demirtas (2004). The correlation matrix was generated randomly as

$$\Sigma \oslash \sqrt{\sigma_{\text{diag}}\sigma_{\text{diag}}^T} \tag{2}$$

with $\Sigma = AA^T \in \mathbb{R}^{10\times 10}$. The entries of A were drawn from a standard normal distribution. The vector $\sigma_{\text{diag}} \in \mathbb{R}^{10}$ refers to the column vector containing the diagonal elements of Σ. The symbol $\oslash$ indicates element-wise divisions, and the square root is also taken element-wise.

Simulation Study 2

For the second simulation study, values from a multivariate normal distribution were drawn using the `mvrnorm` function of the R package **MASS** (version 7.3-54, Venables and Ripley 2002). The correlation matrix was computed as in the first simulation study.

Regarding the alternative approaches in the second simulation study, the following R functions, packages and specifications were used: *BoostingOnly* was implemented using the `gamboost` function in the **mboost** package (version 2.9-8 with `GaussCloglog` family, see Section S1, Hothorn et al. 2022a). We used the same base-learners as for PRT (including the monotonic time base-learner). The optimal value of m_{stop} was determined using the `cvrisk` function in the **mboost** package (five-fold cross-validation). *TreeOnly* was applied using the `ctree` function in the **partykit** package (version 1.2-20, Hothorn et al. 2022b). We controlled the depth of the tree as well the minimum number of observations in the nodes in the same way as for PRT. For *GEE*, the R package **geepack** (version 1.3.9) with the function `geese` was used (Halekoh et al., 2006). The correlation structure was set to "independence". For *Cox*, *Kaplan-Meier* and *Lognormal*, we used the **survival** package (version 3.2-13, Therneau 2021). For *Lognormal*, we used the `survreg` function with the option `dist = "lognormal"`. *BoostedTree* was implemented using the `gbm` function of the R package **gbm** (version 2.1, Greenwell et al. 2020). The interaction depth was set to 2. The optimal m_{stop} was determined by five-fold cross-validation (specifying the `cv.folds` argument in the `gbm` function). The minimum number of observations in the tree base-learner was set to 10. *MOB* was applied using the `mob` function of the **partykit** package (version 1.2-20, following the German breast cancer data example in Zeileis and Hothorn 2015). The control parameters of the tree were the same as in PRT. For *IPCW*, we implemented an inverse-probability-of-censoring-weighted least squares model with log-transformed event times, following the approach by Molinaro et al. (2004). *SRF* was fitted using the `ranger` function of the **ranger** package (version 0.15.1, Wright and Ziegler 2017). The number of variables used as possible split candidates in each node was tuned using five-fold cross-validation.

S5 Results of the second simulation study for $D > 2$

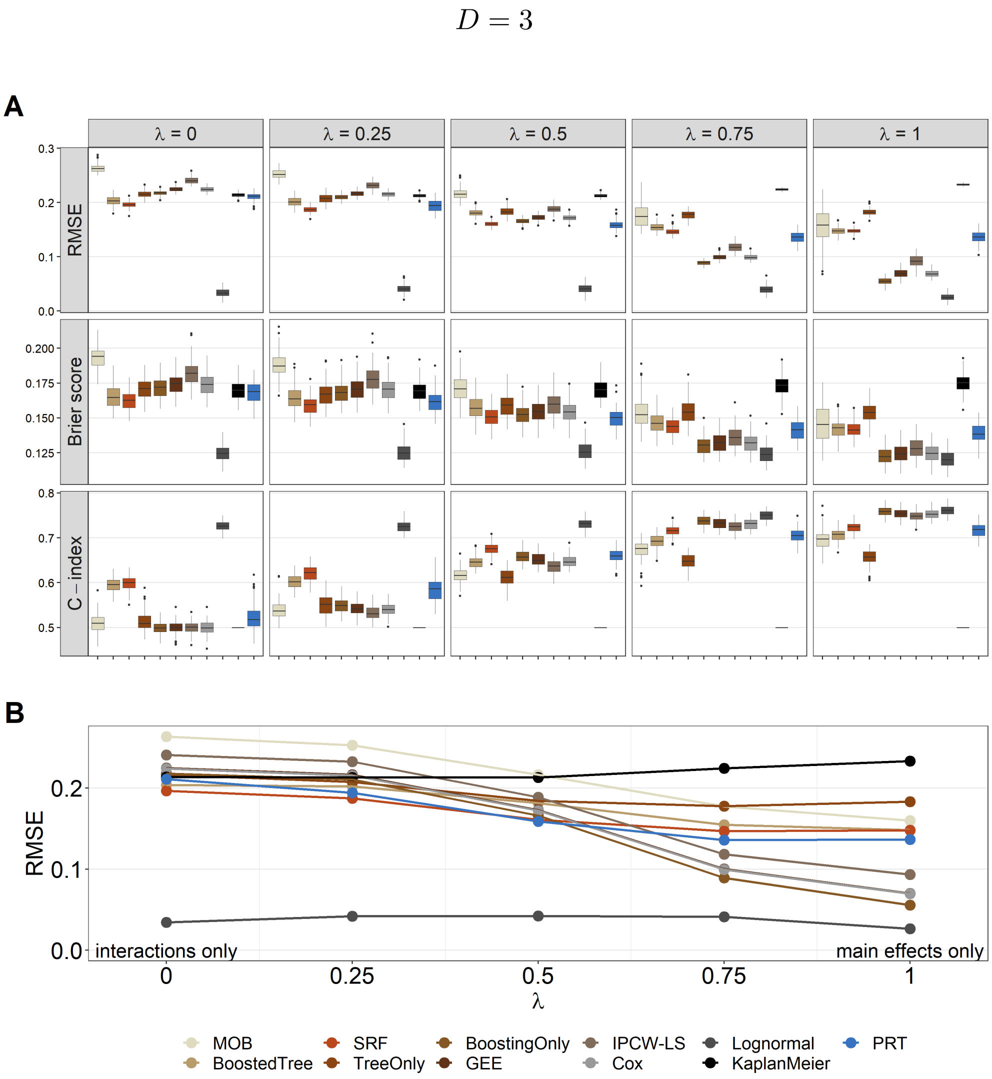

Fig. S3 Results of the second simulation study ($D = 3$, 100 Monte Carlo replications). **(A)** Boxplots of the RMSE values, Brier score values and C-index values, as obtained by evaluating the model fits on the 100 test data sets. **(B)** Mean RMSE values (across the replications). Note that *MOB* did not converge in some of the replications (failure rates = $2\%, 1\%, 2\%, 0\%$, and 1% for $\lambda = 0, 0.25, 0.5, 0.75$, and 1, respectively). The results of these models were excluded from the plots.

$D = 4$

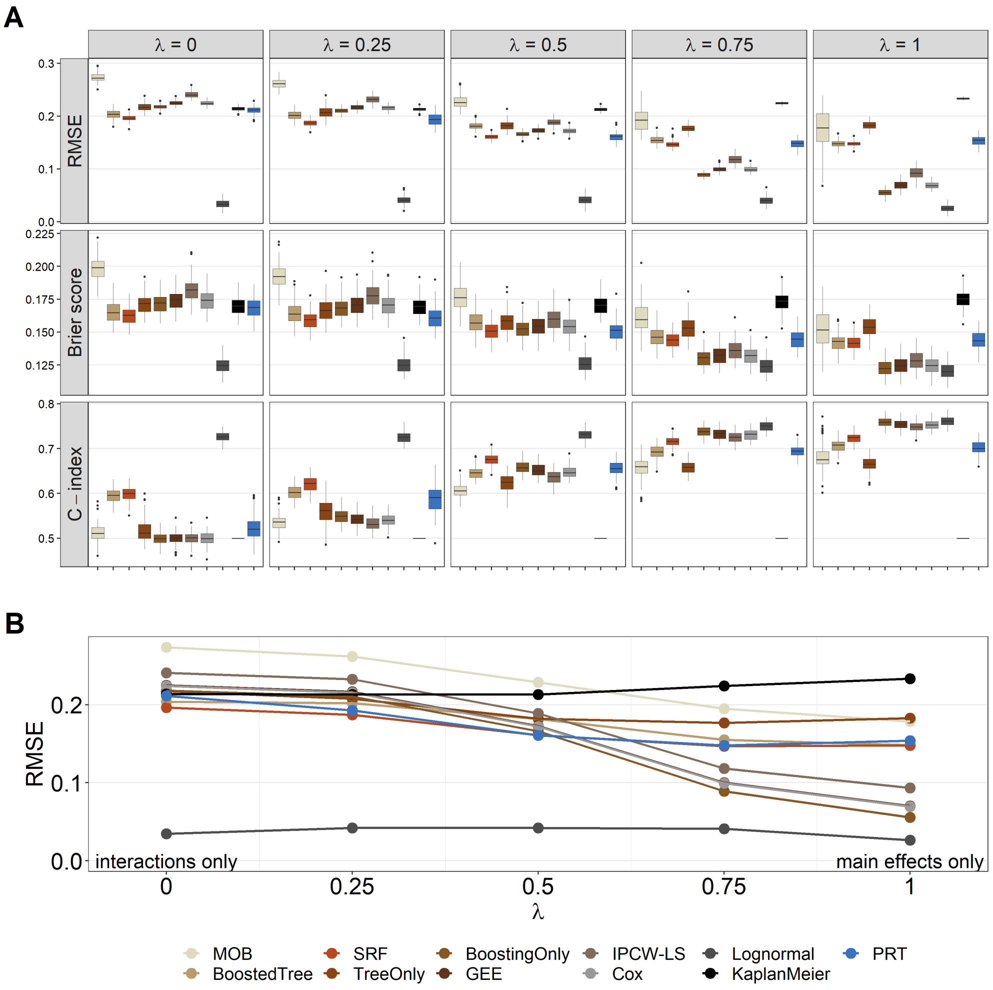

Fig. S4 Results of the second simulation study ($D = 4$, 100 Monte Carlo replications). **(A)** Boxplots of the RMSE values, Brier score values and C-index values, as obtained by evaluating the model fits on the 100 test data sets. **(B)** Mean RMSE values (across the replications). Note that *MOB* did not converge in some of the replications (failure rates $= 2\%, 1\%, 5\%, 1\%$, and 1% for $\lambda = 0, 0.25, 0.5, 0.75$, and 1, respectively). The results of these models were excluded from the plots.

$D = 5$

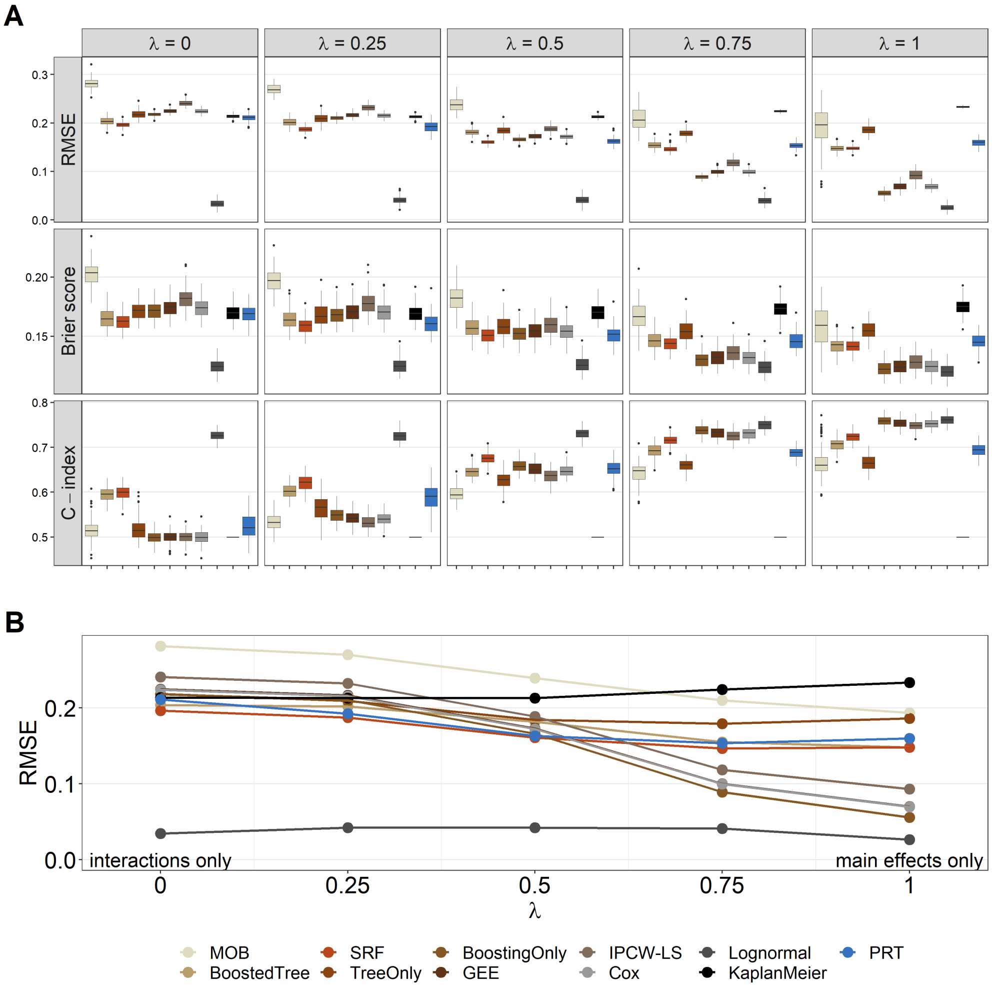

Fig. S5 Results of the second simulation study ($D = 5$, 100 Monte Carlo replications). **(A)** Boxplots of the RMSE values, Brier score values and C-index values, as obtained by evaluating the model fits on the 100 test data sets. **(B)** Mean RMSE values (across the replications). Note that *MOB* did not converge in some of the replications (failure rates = $2\%, 2\%, 5\%, 1\%$, and 1% for $\lambda = 0, 0.25, 0.5, 0.75$, and 1, respectively). The results of these models were excluded from the plots.

S6 Bias of PRT in the second simulation study

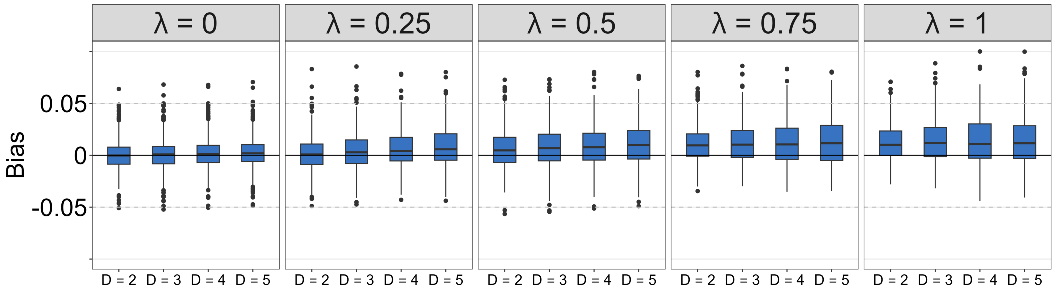

Fig. S6 The boxplots present the bias values obtained from PRT in the second simulation study. For $2 \leq D \leq 5$, PRT resulted in nearly unbiased predictions when λ took small values (i.e., when interaction effects were large). Conversely, the absolute bias increased in the settings with high values of λ (corresponding to small interaction effects). For all tree depths, most of the bias values ranged between -0.05 and 0.05.

S7 Application

Characteristic	Patients (n=3,754)
**age (years)**	
Mean (SD)	53.5 (10.5)
Median [Min, Max]	53.0 [21.0, 86.0]
**BMI** (kg/m^2)	
Mean (SD)	26.3 (5.03)
Median [Min, Max]	25.4 [15.4, 53.4]
**tumor stage**	
pT1	1,552 (41.3%)
pT2	1,929 (51.4%)
pT3	198 (5.3%)
pT4	52 (1.4%)
Missing	23 (0.6%)
**tumor grade**	
G1	176 (4.7%)
G2	1,783 (47.5%)
G3	1,773 (47.2%)
Missing	22 (0.6%)
**lymph node status**	
pN+	2,452 (65.3%)
pN0	1,273 (33.9%)
Missing	29 (0.8%)
**tumor type**	
ductal	3,060 (81.5%)
lobular	419 (11.2%)
other	253 (6.7%)
Missing	22 (0.6%)
**estrogen receptor status**	
ER-	1,252 (33.4%)
ER+	2,481 (66.1%)
Missing	21 (0.6%)
**progesterone receptor status**	
PR-	1,525 (40.6%)
PR+	2,205 (58.7%)
Missing	24 (0.6%)
**HER2**	
HER2-	2,787 (74.2%)
HER2+	883 (23.5%)
Missing	84 (2.2%)
**menopausal status**	
pre	1,565 (41.7%)
post	2,189 (58.3%)
**treatment group**	
control	1,898 (50.6%)
intervention	1,856 (49.4%)

Table S3: Patient characteristics of the SUCCESS-A study population.

References

H. Demirtas. Pseudo-random number generation in R for commonly used multivariate distributions. *Journal of Modern Applied Statistical Methods*, 3:485–497, 2004.

H. Demirtas, R. Allozi, and R. Gao. *MultiRNG: Multivariate pseudo-random number generation*, 2021. URL `https://CRAN.R-project.org/package=MultiRNG`. R package version 1.2.4.

B. Greenwell, B. Boehmke, J. Cunningham, and GBM Developers. *gbm: Generalized boosted regression models*, 2020. URL `https://CRAN.R-project.org/package=gbm`. R package version 2.1.8.

U. Halekoh, S. Højsgaard, and J. Yan. The R package geepack for generalized estimating equations. *Journal of Statistical Software*, 15/2:1–11, 2006.

B. Hofner, A. Mayr, N. Robinzonov, and M. Schmid. Model-based boosting in R: A hands-on tutorial using the R package mboost. *Computational Statistics*, 29:3–35, 2014.

T. Hothorn and A. Zeileis. partykit: A modular toolkit for recursive partytioning in R. *Journal of Machine Learning Research*, 16:3905–3909, 2015.

T. Hothorn, K. Hornik, and A. Zeileis. Unbiased recursive partitioning: A conditional inference framework. *Journal of Computational and Graphical Statistics*, 15:651–674, 2006.

T. Hothorn, P. Bühlmann, T. Kneib, M. Schmid, and B. Hofner. *mboost: Model-Based Boosting*, 2022a. URL `https://cran.r-project.org/web/packages/mboost`. R package version 2.9-7.

T. Hothorn, H. Seibold, and A. Zeileis. *partykit: A Toolkit for Recursive Partytioning*, 2022b. URL `https://cran.r-project.org/web/packages/partykit`. R package version 1.2-16.

A. M. Molinaro, S. Dudoit, and M. J. van der Laan. Tree-based multivariate regression and density estimation with right-censored data. *Journal of Multivariate Analysis*, 90:154–177, 2004.

M. Pohar Perme and M. Gerster. *pseudo: Computes pseudo-observations for modeling*, 2017. URL `https://cran.r-project.org/web/packages/pseudo`. R package version 1.4.3.

T. M. Therneau. *A package for survival analysis in R*, 2021. URL `https://CRAN.R-project.org/package=survival`. R package version 3.2-13.

W. N. Venables and B. D. Ripley. *Modern Applied Statistics with S*. Springer, New York, fourth edition, 2002.

M. N. Wright and A. Ziegler. ranger: A fast implementation of random forests for high dimensional data in C++ and R. *Journal of Statistical Software*, 77:1–17, 2017.

A. Zeileis and T. Hothorn. *Parties, models, mobsters: A new implementation of model-based recursive partitioning in R*, 2015. URL `https://cran.r-project.org/web/packages/partykit/vignettes/mob.pdf`.
